# Affinity‐Based Isolation and One‐Pot Analysis of Extracellular Vesicles from Biofluids Using Phase Separated Zwitterionic Coacervates

**DOI:** 10.1002/advs.202411653

**Published:** 2025-04-15

**Authors:** Francesca Torrini, Roberto Frigerio, Jonathan Garlipp, Philippe Lenzen, Karl Normak, Carolina Paganini, Marina Cretich, Alessandro Gori, Paolo Arosio

**Affiliations:** ^1^ Department of Chemistry and Applied Biosciences, Institute for Chemical and Bioengineering ETH Zurich Zurich 8093 Switzerland; ^2^ Consiglio Nazionale delle Ricerche Istituto di Scienze e Tecnologie Chimiche “Giulio Natta” (SCITEC) Milan 20131 Italy

**Keywords:** extracellular vesicles, isolation, separation, liquid biopsy, zwitterionic coacervates, biofluids, diagnostic analysis

## Abstract

Extracellular vesicles (EVs) hold promise for liquid biopsy and drug delivery applications. However, their heterogeneous nature poses challenges for efficient and selective isolation from complex biofluids. Here, an isolation method based on phase‐separated zwitterionic (ZW) coacervates is developed. These coacervates form over a wide range of pH values and ionic strengths, ensuring compatibility with all biofluids. They exhibit antifouling properties that minimize nonspecific binding, allowing for the selective isolation of EVs from biofluids upon functionalization of the polymer with a suitable affinity probe, as proved here with a membrane‐sensing peptide. This strategy is applied to pull down, concentrate, and release EVs from urine samples with high yields while retaining their structural integrity. This approach effectively separates EVs from lipoproteins, a challenging task for conventional separation techniques. The power of the approach is demonstrated as a preparative step for downstream analysis and as a one‐pot assay to profile EV biomarkers in complex fluids. The latter application, implemented here with flow cytometry, significantly streamlines pre‐analytical workflows. Thus, functionalized ZW coacervates represent an effective strategy for the selective isolation and direct analysis of EVs from complex mixtures, paving the way for advances in large‐scale manufacturing and diagnostics.

## Graphical abstract

This study presents a novel method for affinity‐based isolation and analysis of extracellular vesicles from biofluids using phase‐separated zwitterionic coacervates functionalized with a membrane‐sensing peptide. This technology minimizes nonspecific binding, enabling high‐yield isolation and direct one‐pot biomarker profiling. It significantly streamlines the analytical workflow and shows potential for applications in both large‐scale manufacturing and liquid biopsy.

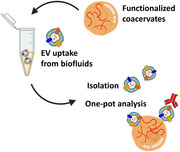

## 1 Introduction

Extracellular vesicles (EVs) are cell‐derived nanoscale particles acting as key players in intercellular communications.^[^ ^1^ ^]^ EVs carry unique molecular fingerprints (e.g., nucleic acids, proteins, lipids, and metabolites) both in their lumen and on their external surface that can specifically report on the state of the parent cells.^[^ ^2^ ^]^ The presence of specific molecular signatures combined with their widespread presence in body fluids (e.g., blood, urine, tear fluid, sweat, and saliva)^[^ ^3^, ^4^ ^]^ makes EVs promising biomarkers, with important implications in noninvasive liquid biopsy for diagnosis and monitoring of physiopathological conditions.^[^ ^5^ ^]^ EVs are also being exploited in materials science as nanocarriers for the delivery of therapeutic payload and cell‐free therapeutics in regenerative medicine.^[^ ^6^, ^7^ ^]^

Despite the burgeoning interest, these promising applications of EVs are currently still hampered by their complex physicochemical properties and their inherent heterogeneity in terms of size, origin, and molecular composition, which complicate their bioprocessing and analytical characterization.^[^ ^8^ ^]^ One of the bottlenecks in translating EVs from bench to bedside lies in the lack of a streamlined and high‐yield isolation process from biofluids and cell cultures. This critical step can hinder the subsequent investigation of EV biological properties and the full realization of their clinical and industrial potential.

Currently, EV isolation is typically performed by ultracentrifugation and/or size‐exclusion chromatography (SEC) along with tangential flow filtration (TFF).^[^ ^7^, ^9^ ^]^ The former isolates EVs of similar densities but of different sizes and compositions, while the latter separates particles according to their size but may result in the isolation of EVs of different densities. As a consequence, both ultracentrifugation and SEC provide good separation yields but are limited in their specificity, allowing impurities with physical properties similar to EVs to co‐isolate with the product. This is a major problem since the secretome contains various particles (e.g., lipoproteins, nucleosomes, exosomes, supermeres, etc.) with similar size and density to EVs.^[^ ^9^, ^10^ ^]^ Moreover, conventional chromatographic methods have historically been developed for single (macro)molecules rather than for multimolecular, nanosized complexes such as EVs, posing challenges in terms of scalability. Novel materials and methods tailored to the characteristics of EVs would improve their isolation from body fluids and cell culture, enhancing their quality when used as nanocarriers and facilitating their analysis when studied for diagnostics.

As an alternative to chromatographic methods, technologies based on precipitation and liquid‐liquid associative/segregative phase separation (LLPS) have been proposed for the purification of biomolecules.^[^ ^11^, ^12^, ^13^, ^14^ ^]^ In this context, we have recently developed programmable zwitterionic copolymers that can self‐assemble via attractive electrostatic forces into polymer‐rich coacervates that are responsive to various environmental parameters such as ionic strength and temperature.^[^ ^15^ ^]^ Moreover, these polymer coacervates offer several advantages over solid materials commonly used in separation technologies. First, due to their liquid‐like nature, coacervates provide a gentle phase that preserves the integrity of analytes during isolation. Second, zwitterionic polymers have pronounced marked anti‐fouling activity, which minimizes non‐specific interactions and allows for the selective recruitment of target molecules into the functionalized coacervates.^[^ ^15^, ^16^ ^]^ Additionally, the technology is scalable and adaptable to different products and/or instrumentation, representing a potential breakthrough in several research areas spanning from bioanalysis, biosensing, and bioprocessing. In the latter field, we have recently demonstrated the potential of positively charged polymers to separate negatively charged (macro)molecules according to the ion exchange principle.^[^ ^17^ ^]^ In clinical applications, however, the use of charged polymeric scaffolds may be limited, mainly due to the need for selective isolation of analytes from complex biofluids.

In this work, we develop zwitterionic coacervates tailored for the specific isolation of EVs from complex mixtures by functionalizing the polymeric scaffolds with specific affinity probes. Specifically, we functionalized the polymer with an amphipathic peptide that targets the peculiar EV phospholipid membrane, rather than binding to specific EV‐associated protein markers, thereby acting as a pan‐vesicular affinity probe.^[^ ^18^ ^]^ This peptide, which belongs to the family referred to as membrane sensing peptides (Bk‐MSP), is derived from bradykinin^[^ ^19^ ^]^ and features a sequence with repetitive motifs combining positive arginine residues, hydrophobic amino acid residues, and hydrophilic spacers^[^ ^20^, ^21^ ^]^ that collectively anchor vesicle membranes. The binding mechanism of MSP leverages the loosened lipid packing of highly tensioned membranes, which favors the insertion of amphipathic peptides into the lipid bilayer. This favors the binding of this class of ligands with nanosized lipid (bio)nanoparticles in the low nanometer range (<200 nm), irrespective of their surface marker profile.^[^ ^18^ ^]^ Bk‐MSP binding to EVs is therefore driven by a cooperative effect given by multiple copies of the peptide in spatial proximity, in contrast to the canonical stoichiometric antibody‐target interaction.

**Figure 1:**
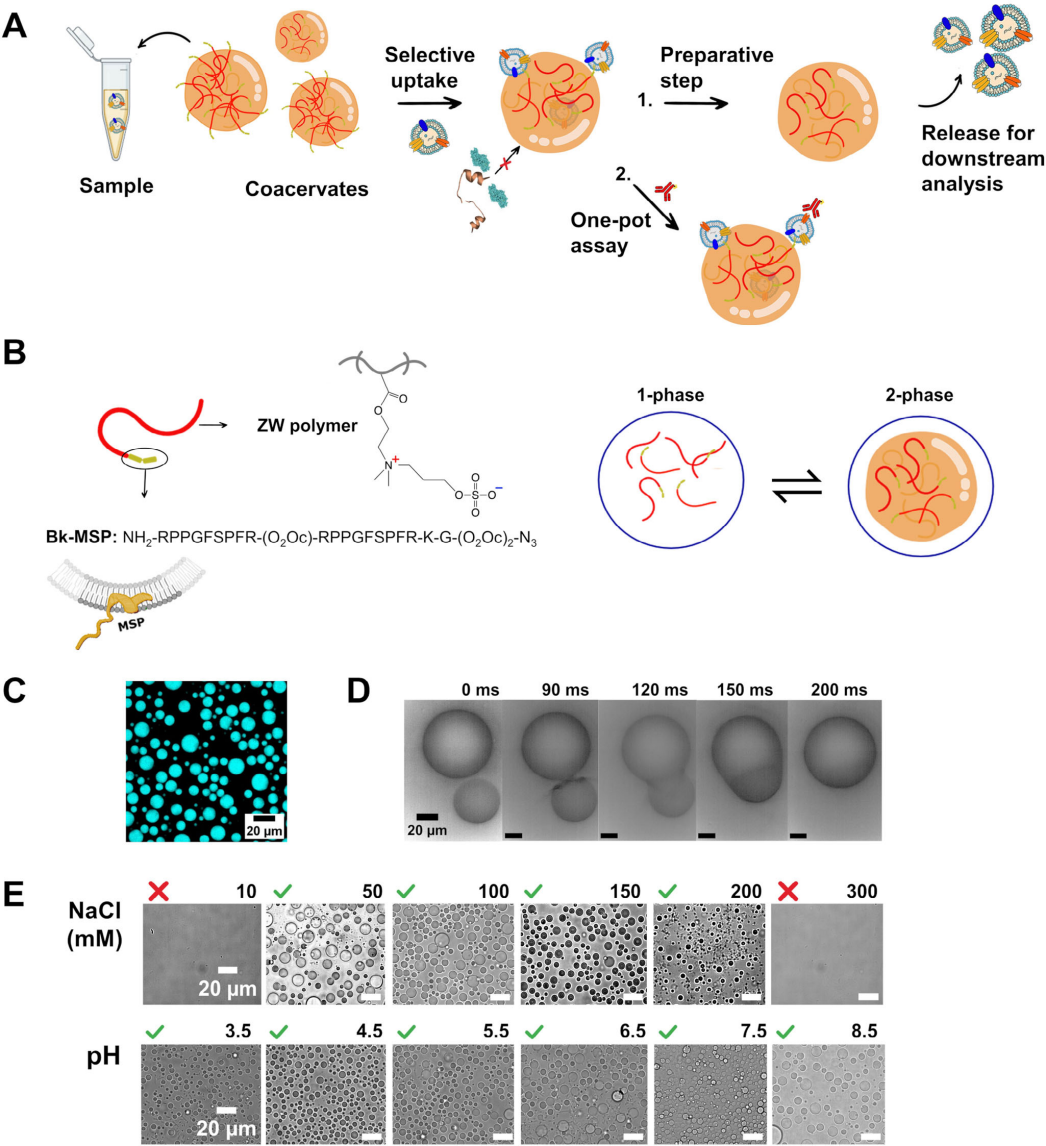
A) Schematic illustration of the two application routes for coacervates investigated in this study: 1) preparative isolation of EVs for downstream analysis; 2) Analysis of EV surface markers by designing a homogeneous “one‐pot assay”. B) Schematic representation of the zwitterionic polymer functionalized with a membrane‐sensing peptide undergoing a liquid‐liquid phase separation process. C) Representative confocal microscopy image of functionalized coacervates formed by 0.8 g L^−1^ polymer with Cy5‐azide. D) Microscopy images showing a fusion event between two liquid‐like coacervates. E) Brightfield microscopy images of 0.8 g L^−1^ polymer solution at constant pH 7 and different salt concentrations (top panels); at constant salt concentration (100 mM) and different pH values (bottom panel).

We illustrate the power and versatility of the affinity coacervate‐based isolation strategy in two application routes (**Figure** **1**A): first, as a preparative step for downstream analysis, and second, as part of an analytical “one‐pot assay” for the analysis of EV biomarker signatures in complex fluids.

## 2 Results and Discussion

### 2.1 Design of Functionalized Zwitterionic Coacervates for EV Affinity Capture

The zwitterionic copolymer contains the monomer sulfobetaine methacrylate (ZB) (Figure 1B), which induces an enthalpy‐driven phase separation promoted by attractive electrostatic interactions mediated by the paired charges.^[^ ^15^ ^]^ ZB was co‐polymerized with the monomer N‐(3‐sulfopropyl)‐N‐methacroyloxyethyl‐N,N‐dimethylammonium betaine (SB), which further modulates the solubility of the peptide, according to the degree of polymerization (DP) DP_ZB_ = 140 ZB and DP_SB_ = 60 (see details in the experimental section and Supporting Information, including characterization in Figure S1A, Supporting Information). This zwitterionic polymer scaffold was conjugated to the Bk‐MSP peptide using a copper‐free azide/DBCO bioorthogonal click reaction (Figure 1B; see further details in the experimental section).

We characterized the functionalization yield by size‐exclusion chromatography (SEC) coupled to an in‐line fluorescence detector (see chromatogram in Figure S1B, Supporting Information), obtaining a functionalization efficiency of ≈80%. The functionalized polymer was stored as a homogeneous solution at a high salt concentration (1.2 m NaCl), which screens the attractive electrostatic interactions and prevents phase separation. Decreasing the ionic strength of the solution leads to the formation of coacervates (Figure 1C), which are liquid‐like, as evidenced by the rapid fusion events observed on a millisecond time scale (Figure 1D). Importantly, phase separation remains consistent over a wide range of salt concentrations (10–200 mM) and pH values (3.5–8.5) (Figure 1E; Figure S1C,D and S2, Supporting Information), encompassing physiological conditions.

Such versatility is a key requirement for the use of the material in the isolation and analysis of target (bio)molecules from complex matrices.

### 2.2 Uptake and Release of Liposomes

**Figure 2:**
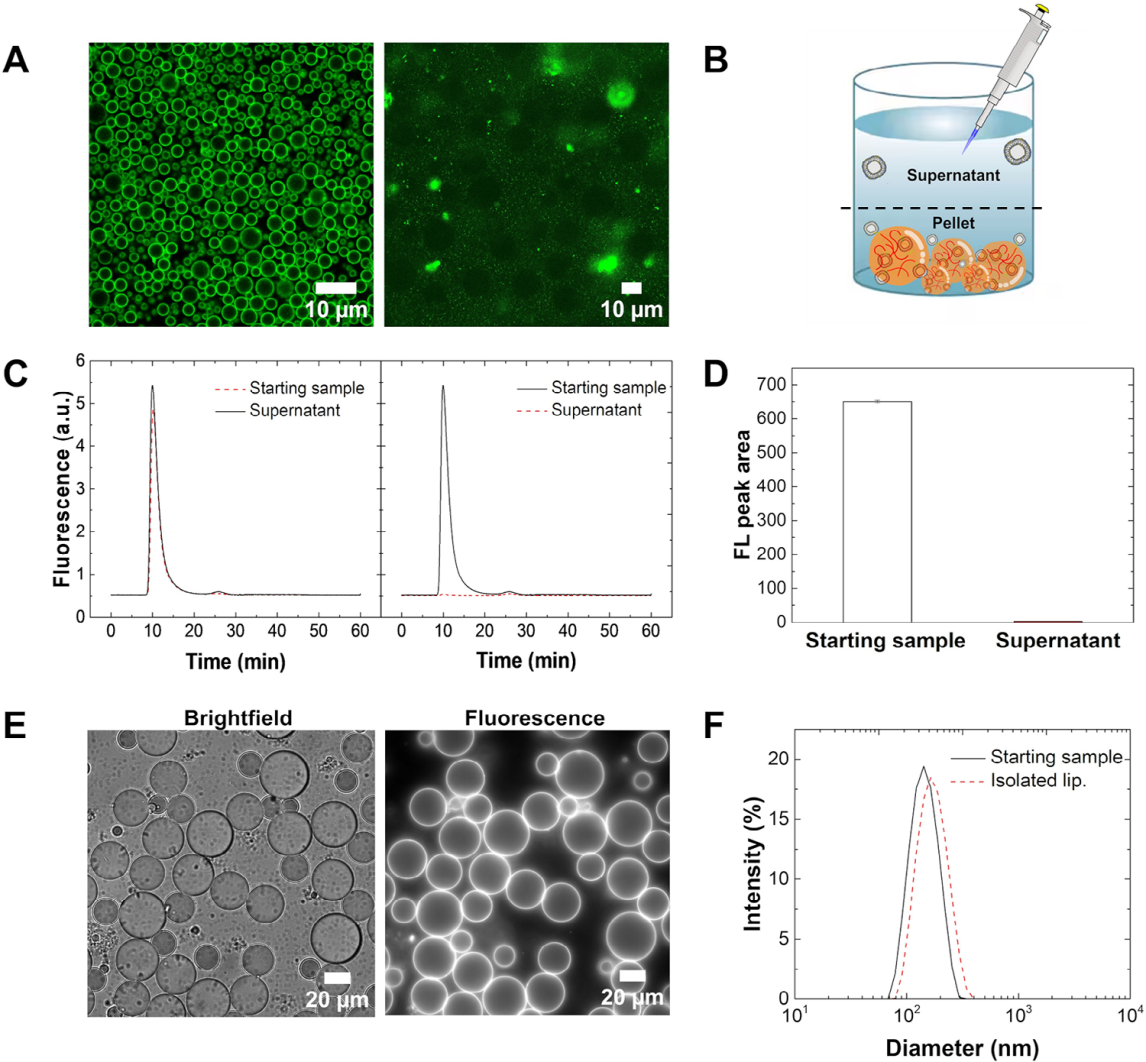
A) Confocal microscopy images of DiO‐labeled liposomes (DiO‐Lip.), used here as a model of EVs, in the presence of functionalized (left panel) and non‐functionalized (right panel) coacervates. B) Sketched representation of how the recruitment of DiO‐Lip. Into coacervates was evaluated. Dilute and dense phases were separated by centrifugation and the concentration of DiO‐Lip. The supernatant was measured using a combination of SEC and in‐line fluorescence detection. C) SEC chromatogram of the starting liposome sample (5.5 × 10^9^ particles mL^−1^) and the dilute phase after recruitment by functionalized (right) and non‐functionalized (left) coacervates (2.5 g L^−1^). D) Area under the peak corresponding to DiO‐Lip. (FL_mean_ ± SD, *n* = 3) in the SEC chromatograms of the original sample and of the dilute phase after separation from the dense phase. E) Brightfield and fluorescence microscopy images of the coacervates with DiO‐Lip. Enriched in the dense phase after centrifugation. F) Size distribution of the starting liposome sample and the liposomes recovered from the coacervates as measured by DLS.

We preliminarily tested the ability of the Bk‐MSP‐functionalized coacervates to recruit vesicles using DiO‐labeled liposomes with an average hydrodynamic diameter of 109 ± 4 nm (hereafter referred to as DiO‐Lip.; see characterization in Figure S3, Supporting Information). In our work, liposomes are a suitable model of EVs for preliminary experiments as the Bk‐MSP affinity probe targets the lipid membrane. We mixed DiO‐Lip. at 3.3 × 10^10^ particles mL^−1^ (as measured by nanoparticle tracking analysis, NTA, see Figure S3, Supporting Information) with 0.8 g L^−1^ of functionalized polymer in 100 mM NaCl solution at pH 7. After 1 h of incubation at room temperature in a 384‐well plate pre‐coated with BSA, we analyzed the recruitment of DiO‐Lip. In the functionalized coacervates by optical and confocal microscopy. The confocal image (**Figure** **2**A, left image) shows colocalization of the liposomes at the interface of the coacervates, demonstrating successful uptake. The localization of DiO‐Lip. The rim of the condensates is consistent with previous findings observed with positively charged coacervates^[^ ^17^ ^]^ where liposomes were recruited by electrostatic interactions. This behavior is due to the higher hydrophilicity of the polymer‐liposome complex compared to the unbound polymer that forms the core of the coacervate.

Control experiments with non‐functionalized coacervates confirmed the absence of liposome uptake (Figure 2A, right image). Moreover, coacervates functionalized only with a DBCO click handle and with different ligands (namely, azide‐PEG3‐biotin and Cy5‐azide) were not able to substantially recruit liposomes (Figure S4, (Supporting Information) left panel). Finally, to rule out artifacts related to the possible presence of free dye in the sample, we mixed DiO with both functionalized and nonfunctionalized coacervates, confirming the absence of non‐specific recruitment of free dye (Figure S4, (Supporting Information) right panel). Overall, these control experiments confirm that liposome recruitment occurs only in the presence of peptide‐functionalized coacervates.

To quantify the uptake of liposomes into the coacervates, we measured the amount of DiO‐Lip. The dilute phase separates the dense and dilute phases by centrifugation and analyzing the supernatant by SEC combined with an in‐line fluorescence detector.^[^ ^22^ ^]^ The amount of liposomes was estimated by integrating the area corresponding to the peak of DiO‐Lip. In the chromatogram (Figure 2C) and normalizing with respect to the area of a reference sample, which was not incubated with the coacervates (Figure 2D). We obtained 99% uptake of DiO‐Lip. In functionalized coacervates (Figure 2C, left) and no uptake in control non‐functionalized coacervates (Figure 2C, right). As an orthogonal method, we further analyzed the polymer‐enriched coacervates by brightfield and fluorescence microscopy (Figure 2E). The images confirmed the presence of DiO‐Lip. At the interface of the coacervates after the removal of the dilute phase.

We next tested the selectivity of liposome recruitment in the presence of competing molecules by adding 500 nM of labeled IgG, GFP, and BSA in 10 mM PBS at pH 7.4. All molecules have a negative net charge at neutral pH. Brightfield and fluorescence microscopy analysis (Figure S5A, Supporting Information) showed that these molecules were excluded by the coacervates. The lack of recruitment was further confirmed by quantifying the concentration of the molecules in the dilute phase after the removal of the coacervates (Figure S5B, Supporting Information). Taken together, these findings robustly confirm that the Bk‐MSP‐based coacervates selectively recruit the target vesicles.

The liposomes can be released from the coacervates by exploiting the stimulus‐responsiveness of phase separation and the dependence of the peptide‐vesicle interactions on ionic strength. We dissolved the coacervates by adding 600 mM of NaCl and 500 mM MgCl_2_. This increase in salt concentration leads to the simultaneous dissolution of the dense phase and the release of EVs from the affinity ligand.^[^ ^20^ ^]^ The released liposomes were analyzed by dynamic light scattering (DLS) (Figure 2F). The size distributions of the liposomes before and after isolation are very consistent, confirming the absence of vesicle degradation during the gentle assembly and disassembly of the coacervates. Of note, the polymer can be also easily separated from the vesicles by an additional size exclusion fractionation step and recycled.

### 2.3 Isolation of EVs from Urine

**Figure 3:**
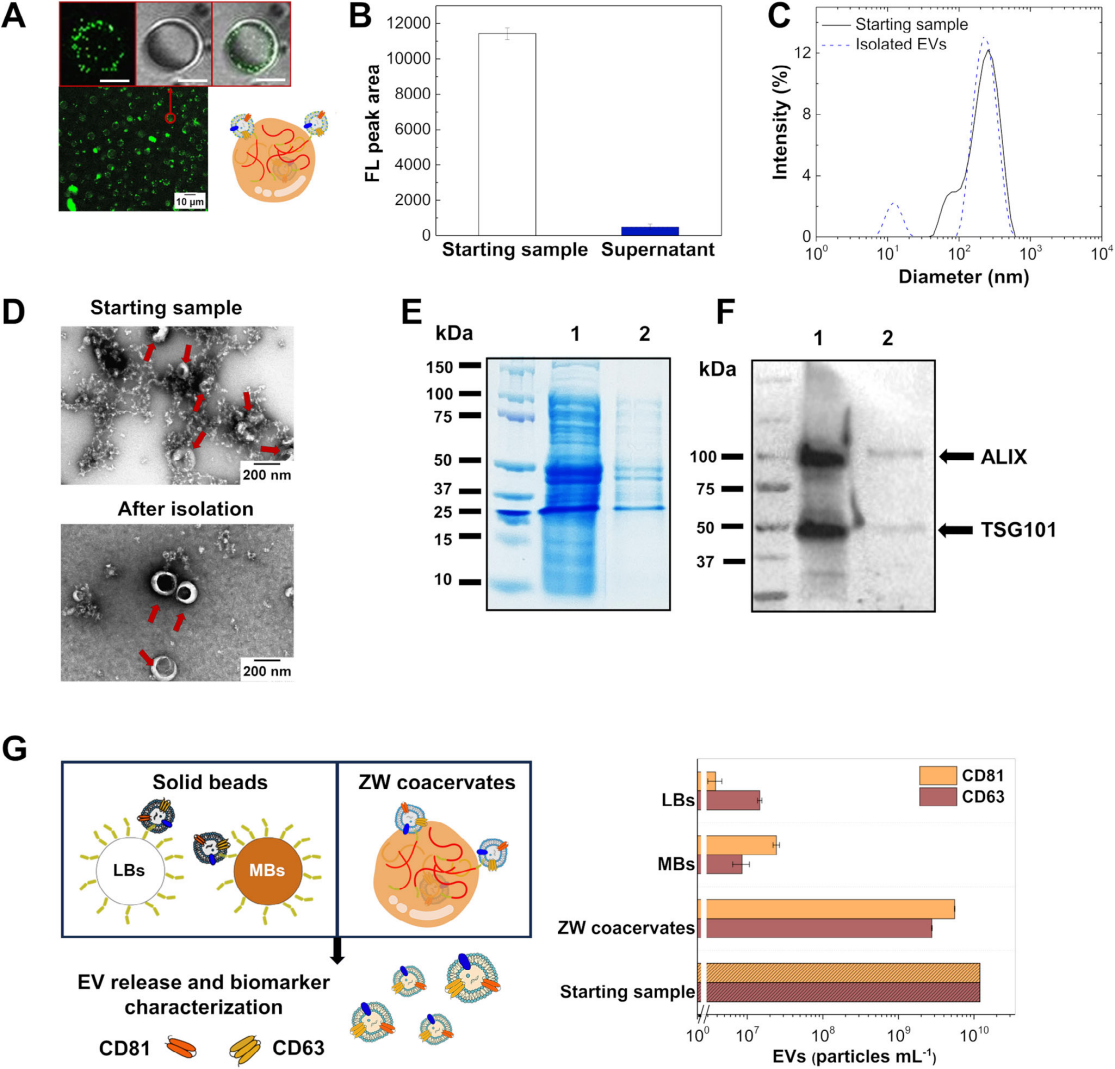
Isolation of EVs from HEK 293‐F cells spiked in artificial urine: A) Confocal microscopy image of 9 × 10^8^ particles mL^−1^ DiO‐EVs spiked in artificial urine with 3.3 g L^−1^ functionalized coacervate. The zoomed panel shows, from left to right, confocal, brightfield, and merged images (scale bar: 10 µm). B) Amount of EVs in the dilute phase measured by SEC coupled with fluorescence detection, before and after incubation of 1.2 × 10^10^ particles mL^−1^ EVs with coacervates (2.7 g L^−1^). The bars report the fluorescence intensity (FL_mean_ ± SD, *n* = 3) of the area under the peak corresponding to EVs in the SEC chromatograms. C) Size distribution as measured by DLS of the starting sample (black line) and the isolated EVs (dashed blue line). D) TEM micrographs of the starting sample and the isolated EVs, which are indicated by red arrows. E) Polyacrylamide SDS‐PAGE coupled with Coomassie staining: lanes 1 and 2 refer, respectively, to the initial sample and to the isolated EVs after dissolution of the coacervates at high salt (10 mM PBS at pH 7.4 with 600 mM NaCl and 500 mM MgCl_2_). F) Western blot analysis of the internal EV signatures ALIX and TSG101: lanes 1 and 2 refer to the original samples and the EVs isolated and released from the functionalized coacervates, respectively. G) Schematic representation of two different isolation methods, one based on solid beads consisting of latex (LBs) and iron oxide (MBs) (left), and the other based on zwitterionic (ZW) liquid‐like coacervates (right). The isolated EVs were captured by anti‐CD81‐beads and characterized by flow cytometry after staining CD63 and CD81 biomarkers. Bars represent the number of EVs (particles mL^−1^) recovered from the coacervates (*n* = 3) and the beads compared to the starting sample (1.2 × 10^10^ particles mL^−1^). A significant difference was observed between the two approaches (ZW coacervates versus MBs, ZW coacervates versus LBs) (*t*‐test, *p* < 0.01).

Having demonstrated the approach with liposomes, we next applied the technology to isolate EVs from urine. First, we established a model of a real sample by mixing EVs derived from HEK 293‐F cells (see characterization details in Supporting Information) at a final concentration of 9×10^8^ particles mL^−1^ in artificial urine with 3.3 g L^−1^ of functionalized polymer. EV uptake was promoted by raising the pH of the solution to 10 with sodium hydroxide,^[^ ^23^ ^]^ followed by 2‐hour incubation in a 384‐well plate under static conditions. We note that, as shown in Figure 1E, phase separation of the zwitterionic coacervate is minimally affected by changes in pH and coacervates can be observed even at this high pH value. The pulled‐down and released EVs from the Bk‐MSP‐functionalized coacervates were analyzed by combining confocal microscopy, SEC, DLS, and transmission electron microscopy (TEM). Confocal microscopy images acquired by staining EVs with DiO confirmed the presence of binding (**Figure** **3**A).

As control experiments, we analyzed the non‐functionalized polymer in the presence of DiO‐EVs, as well as samples containing the free dye mixed with both functionalized and nonfunctionalized polymers (Figure S5C, Supporting Information). Overall, this set of experiments confirmed the presence of binding only between EVs and functionalized coacervates. Uptake was further confirmed by analyzing the EV concentration in the dilute phase by SEC coupled with fluorescence detection (Figure 3B), after separating the dense and dilute phases by centrifugation. In this case, ≈90% of the EVs from HEK 293‐F cells spiked in artificial urine were recruited by the functionalized coacervates (Figure 3B).

EVs were released from the coacervates upon increasing the ionic strength of the solution (600 mM NaCl and 500 mM MgCl_2_), and the integrity of the isolated EVs was verified by DLS analysis (Figure 3C). This result was further corroborated by TEM analysis (Figure 3D), which showed that EVs preserved a round (“cup‐shaped”) morphology with diameters ≈40–200 nm throughout the process and did not show any morphological change upon isolation.^[^ ^24^, ^25^ ^]^

Moreover, the TEM image of the starting sample revealed a significant presence of contaminants, which were substantially reduced after the elution of the captured EVs from the coacervates. To assess the purity of the isolated EVs, we further performed an SDS‐polyacrylamide gel electrophoresis (SDS‐PAGE), followed by Coomassie blue staining (Figure 3E). The results showed that the isolated EVs contained significantly lower levels of contaminants. The integrity of the EVs was confirmed by Western blot analysis, which detected the presence of ALIX (96 kDa) and TSG101 (50 kDa), proteins involved in exosome biogenesis (Figure 3F).^[^ ^26^ ^]^

The liquid‐like nature of coacervates and their stimulus‐responsiveness toward changes in the environment represent important advantages over traditional heterogeneous bead‐based affinity assays. In particular, they offer the opportunity to streamline the preparative steps of biological samples by avoiding intensive washing steps and the need for specific equipment (e.g., magnetic tools). To illustrate this concept, we compared our homogeneous coacervate‐based process with conventional bead‐based affinity assays (Figure 3G). We used two types of commercially available carboxyl‐derivatized beads, i.e., magnetic silanized iron oxide‐ and latex‐based beads (Figure 3G). EVs (1.2 × 10^10^ particles mL^−1^) spiked in artificial urine were isolated in parallel using both the coacervates and the commercial beads (Figure 3G, left scheme), following the same experimental procedure in terms of functionalization and incubation time (see experimental section). The coacervate and bead pellets were then suspended in 600 mM NaCl and 500 mM MgCl_2_ to trigger the release of EVs from the affinity probe.^[^ ^20^ ^]^ The number of isolated EVs was quantified in terms of transmembrane biomarker expression (CD81 and CD63) using a well‐established bead‐assisted flow cytometry method (see experimental section and Supporting Information).^[^ ^17^ ^]^ The assay involves capturing a CD81 EV‐positive subpopulation on anti‐CD81 beads, followed by staining with anti‐CD63 and anti‐CD81 antibodies (Abs). The amount (of particles mL^−1^) of EV isolated and released from the peptide was evaluated using calibration curves generated for each biomarker (Figure 3G, right graph). As a result, the coacervate‐based assay showed a 2‐order‐of‐magnitude increase in EV yield compared to the bead‐based assays, with recoveries of 50% and 28% for CD81‐ and CD63‐positive EVs, respectively, from the starting sample.

**Figure 4:**
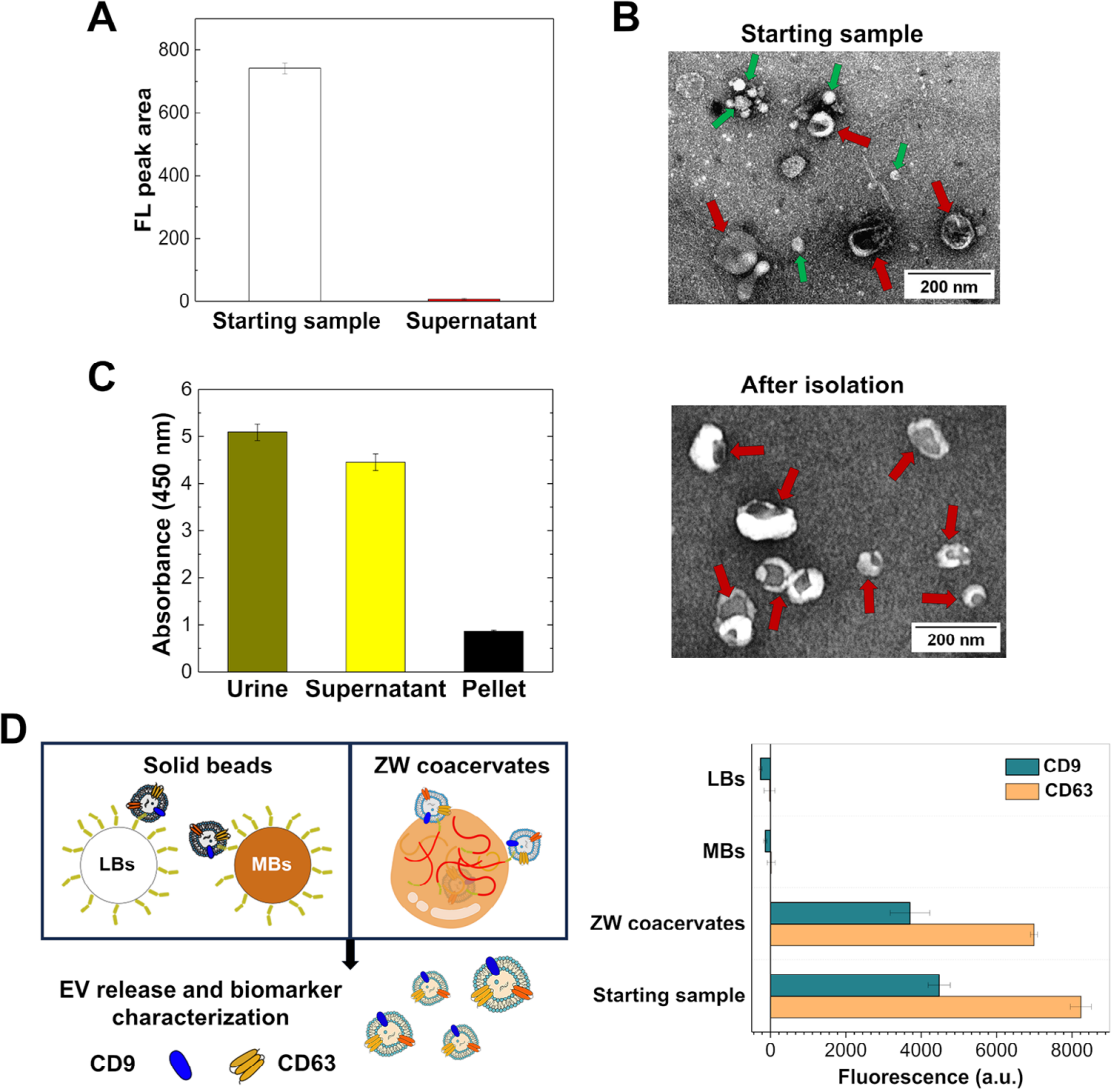
Isolation of EVs from human urine: A) Amount of urinary EVs (starting sample was diluted 1:5) in the dilute phase measured by SEC coupled with fluorescence detection, before and after incubation with 2.7 g L^−1^ coacervate. Bars report the fluorescence intensity (FL_mean_ ± SD, *n* = 3) of the area under the peak corresponding to EVs in the SEC chromatograms. Urinary EVs were labeled with anti‐CD9‐FITC Ab (0.6 nM). B) TEM micrographs comparing the starting urine sample with the isolated urinary EVs (indicated by red arrows). Green arrows in the starting sample indicate the presence of lipoproteins. C) ELISA analysis of the amount of lipoprotein(a) in the original urine sample, in the dilute phase (supernatant) after separation from the dense phase, and in the solubilized dense phase (pellet) containing isolated EVs. The bar graph shows the mean absorbance at 450 nm value from triplicate samples. D) Schematic representation of two different isolation methods, one based on solid beads consisting of latex (LBs) and iron oxide (MBs) (left) and the other based on zwitterionic (ZW) liquid‐like coacervates (right). The bar graph indicates the MFI_mean_ ± SD (*n* = 3) associated with the tetraspanins (CD63 and CD81) present on EVs after being isolated and released by the polymer coacervates and beads, compared to the EV amount in the initial sample (for all beads and coacervates, the isolated EVs were resuspended in half of the original volume). A significant difference was observed between the two approaches (ZW coacervates versus MBs, ZW coacervates versus LBs) (*t*‐test, *p* < 0.01).

Next, we applied the zwitterionic coacervates to isolate EVs from human urine, using the same experimental approach described previously. We measured the concentration of EVs remaining in the dilute phase by SEC coupled with fluorescence detection. For this analysis, we stained EVs with anti‐CD9‐FITC antibody to minimize the contribution to the signal from potential impurities co‐eluting with EVs (e.g., lipoproteins). The SEC profiles (**Figure** **4**A) show the presence of the EV‐anti‐CD9 Ab complex in the starting sample and its absence in the dilute phase of the coacervate sample, confirming the recruitment of EVs in the dense phase. We confirmed these results by TEM microscopy analysis, which showed the presence of EVs in the starting sample, their integrity post‐isolation, and a negligible amount of impurities in the isolated EVs compared to the original sample (Figure 4B). In particular, the TEM image of the isolated sample showed a reduced amount of bright spots, which likely correspond to lipoproteins or protein aggregates.^[^ ^27^ ^]^ Following this observation, we quantified the removal of lipoproteins, which are a major source of co‐isolates in EV preparations from biological fluids.^[^ ^28^, ^29^ ^]^ In particular, lipoprotein (Lp) A, an atherogenic lipoprotein particle composed mainly of apolipoprotein (apo) A, can be excreted in urine under certain pathological conditions^[^ ^30^ ^]^ such as glomerulonephritis, diabetic nephropathy, and amyloidosis.^[^ ^31^ ^]^ Lipoproteins pose significant challenges in terms of removal and separation using traditional techniques such as ultracentrifugation and SEC.^[^ ^28^ ^]^ Lipoproteins are expected to have a very low affinity for the peptide‐functionalized coacervates compared to EVs due to their lipid composition, which is characterized by a lower phosphatidylserine (PS) content.^[^ ^32^ ^]^ We measured the amount of Lp A before and after isolation using a commercial ELISA kit. As shown in Figure 4C, the absorbance value measured for the original urine (Lp A content > 1.1 µg mL^−1^ Figure S5D, Supporting Information) was comparable to the dilute phase obtained after removal of the dense phase by centrifugation, indicating that most lipoproteins are excluded by the coacervates. This result was confirmed by the low absorbance value measured in the dense phase after dissolution, which showed an Lp A depletion of more than 83% compared to the initial sample.

As with the synthetic urine experiments, we compared the yield of EV recovery from human urine using coacervates and conventional solid beads. EVs were detected with antibodies against CD63 and CD9, which are common biomarkers for EV identification in urine samples.^[^ ^26^, ^33^ ^]^ The presence of CD63 and CD9 in our samples was verified by flow cytometry (see details in the experimental section and in Supporting Information). In contrast to the previous experiment performed with EVs from HEK 293‐F cells (Figure 3G), the presence of nanosized contaminants in human urine can affect the evaluation of the particle concentration. Therefore, in this case, the amount of EVs is estimated from the fluorescence signal (MFI_mean_) associated with the anti‐CD9 and anti‐CD63 Abs (Figure 4D, right panel). For the coacervates, we obtained a MFI_mean_ corresponding to 84% of the starting “unprocessed” sample, confirming the higher yield achieved by our coacervate‐based approach compared to the solid bead strategy, which failed to isolate EVs in this experiment. These results can be partially attributed to the larger surface area of the ZW coacervate population, which can dynamically grow to ≈530 µm^2^, far exceeding the average 60 µm^2^ surface area of solid beads, thereby favoring the EV binding to the probe.

### 2.4 “One‐Pot” Isolation and Analysis of EVs with Coacervates

In addition to the preparative step for downstream analysis, the ability of our coacervates to selectively localize and concentrate EVs can also be used to profile EV‐associated markers (e.g., tetraspanins) after appropriate staining (Figure 1A). We implemented this concept of “one‐pot” analysis by analyzing our stained coacervates on a conventional flow cytometer, a technique commonly available in research and clinical laboratories. Albeit conceptually similar to standard bead‐based assays for EV characterization,^[^ ^34^ ^]^ our one‐pot assay based on liquid coacervates can streamline the preparative step, thereby reducing time, cost, and sample handling. Moreover, the antifouling properties of the zwitterionic coacervates minimize non‐specific adsorption, eliminating the need for further passivation steps typically required for solid surfaces.

**Figure 5:**
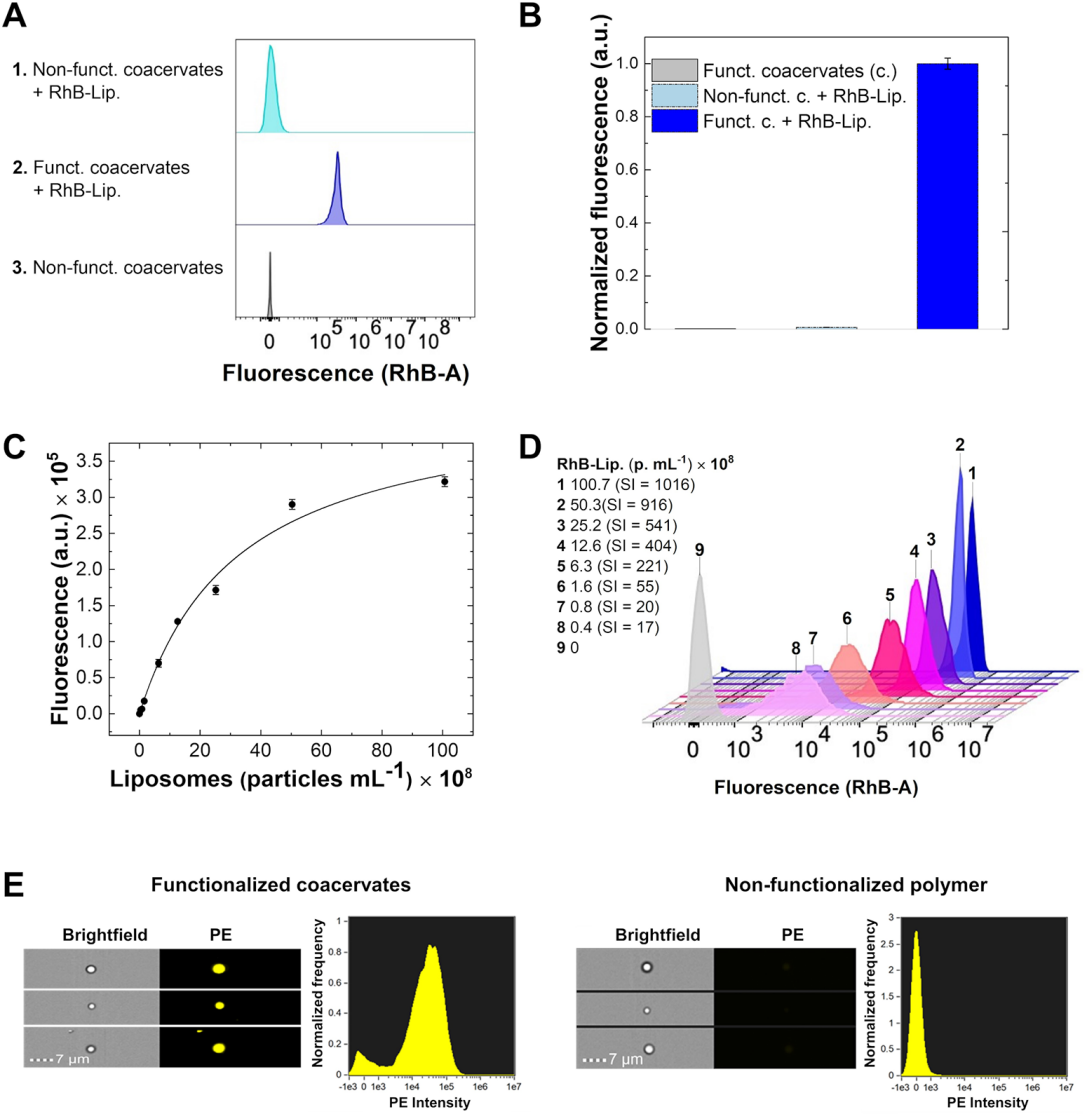
A) Representative flow cytometry histograms of samples containing non‐functionalized polymer with and without RhB‐Lip., and functionalized polymer with RhB‐Lip. All samples were measured at a fixed concentration of polymer (0.8 g L^−1^) and analyte (3 × 10^9^ particles mL^−1^). B) The bar chart shows the normalized mean values of the median fluorescence intensity (MFI) recorded in triplicate (MFI_mean_ ± SD) for each sample. C) Fluorescence intensity measurements for different RhB‐Lip. Concentrations ranging from 3.9 × 10^7^ to 1.0 × 10^10^ particles mL^−1^. The stain index (SI) is defined as the difference between the mean MFI of the positive and negative populations divided by twice the standard deviation of the negative population (SI = MFI_mean_pos – MFI_mean_neg/2 × SD_neg_). SI was calculated for each concentration, showing that SI values increase proportionally with the concentration of the stained analyte. D) Linear fitting of the mean MFI response to different concentrations of RhB‐Lip in buffer, where each point represents the average of three independent measurements (MFI_mean_ ± SD). The blank MFI was subtracted from the entire data set. E) Representative brightfield and fluorescence images (PE) of coacervates and single‐color intensity histograms. The right and left panels refer to non‐functionalized and functionalized coacervates, respectively, with RhB‐Lip (3 × 10^9^ particles mL^−1^).

We first tested this approach with liposomes stained with Rhodamine B (RhB‐Lip.). As expected, the mean median fluorescence signal (MFI_mean_) of the functionalized coacervates was significantly higher in the presence of RhB‐Lip. Compared to control samples that either lacked liposomes or consisted of non‐functionalized coacervates (**Figure** **5**A,B). We assessed the dose‐response relationship by measuring the signal corresponding to increasing RhB‐Lip. concentrations, ranging from 3.9 × 10^7^ particles mL^−1^ to 1.0 × 10^10^ particles mL^−1^ (Figure 5C,D).

The signal responded linearly to increasing concentrations of RhB‐Lip. till 4 × 10^9^ particles mL^−1^. The affinity calibration curves were fitted with a Langmuir model (correlation coefficient, R^2^ = 0.99) by applying the following equation FL = (FL_max_ × [RhB‐Lip.]/(EC_50_ + [RhB‐Lip.]), where FL_max_ (4.4 ± 0.3) is the maximum response and EC_50_ (33 ± 5 particle mL^−1^) corresponds to the liposome amount (particles mL^−1^) that gives 50% of the maximum response. The assay demonstrated excellent repeatability, expressed as an averaged coefficient of variation (_av_CV% = SD/mean) of 2.7%. The limit of detection (LOD ± SD) and the limit of quantification (LOQ ± SD) were 1.1 × 10^5^ ± 1.8 × 10^4^ particles mL^−1^ and 3.5 × 10^5^ ± 6.0 × 10^4^ particles mL^−1^, respectively. In the case of fluorescent liposomes, flow cytometry imaging allows real‐time visual confirmation of detection events by examining coacervates in brightfield and fluorescence images (Figure 5E). For functionalized coacervates in the presence of RhB‐Lip. at 4.6 × 10^9^ particles mL^−1^, we observed an overlap between the fluorescence signal (PE channel) and brightfield images (Figure 5E), confirming the localization of RhB‐Lip. within the coacervates. The fluorescent signal was absent in control non‐functionalized coacervates (Figure 5E).

**Figure 6:**
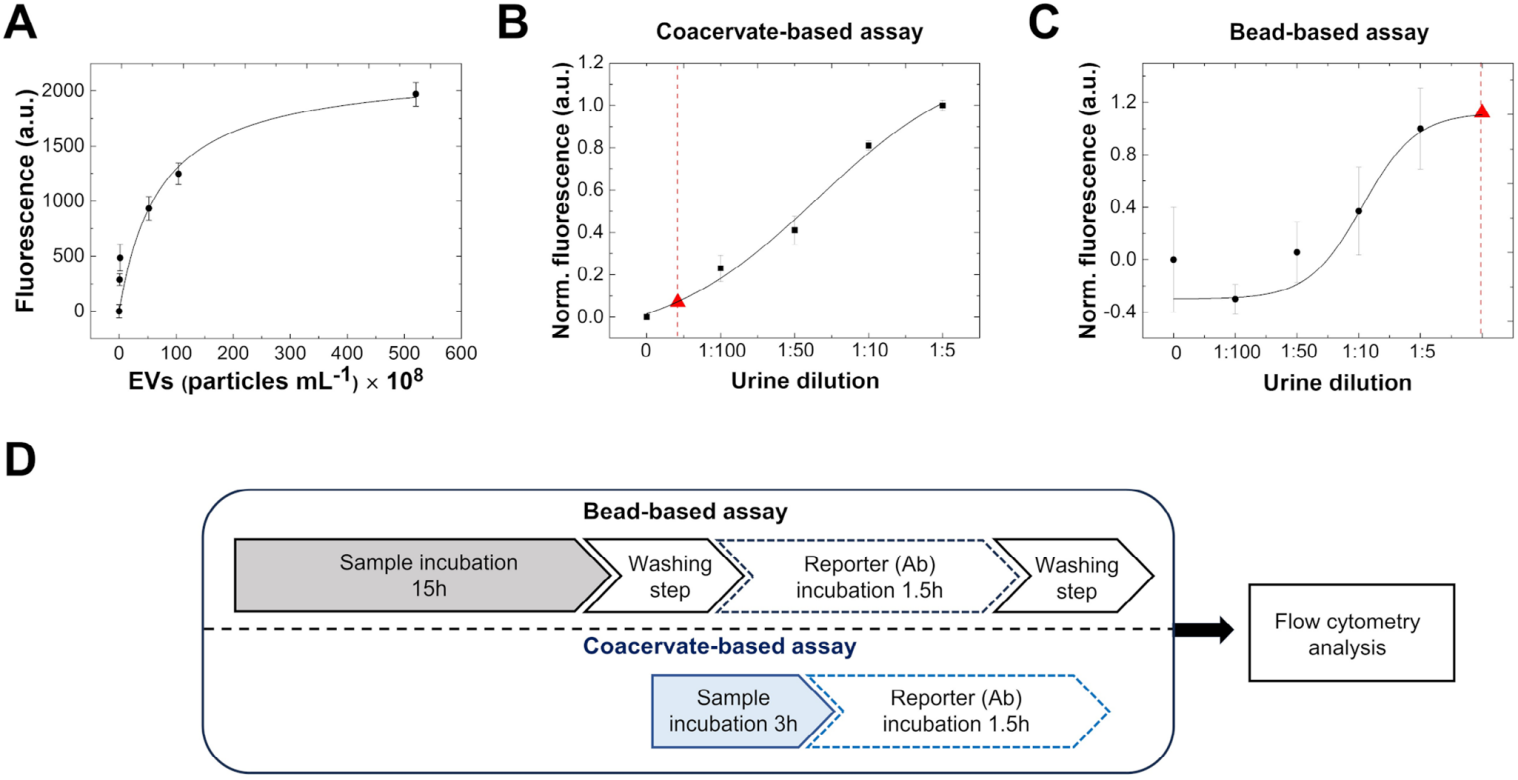
A) Fluorescence signal of functionalized coacervates in the presence of increasing concentrations of EVs from HEK‐293F spiked into artificial urine, ranging from 8.1 × 10^7^ to 5.2 × 10^10^ particles mL^−1^, labeled with anti‐CD81 antibody as the fluorescent signal reporter. B, C) Dilution curve for the “one‐pot” analysis of a human urine sample. EVs were detected with anti‐CD9 Ab using the coacervate‐based assay (B) and with the beads (MBs)‐based assay (C). The blank MFI_mean_ (without urine) was subtracted from all samples. D) Schematic comparison of the pre‐analytical workflows required for the analysis with conventional flow cytometry using the bead‐based assay and the coacervate‐based assay.

After validating the approach with liposomes, we applied this approach to detect EVs from HEK 293‐F cells spiked in artificial urine using an anti‐CD81 antibody as a fluorescent reporter. We established a calibration curve over a range of EV concentrations from 8.1 × 10^7^ to 5.2 × 10^10^ particles mL^−1^ (**Figure** **6**A), achieving a LOD of 5.0 × 10^8^ particles mL^−1^, a LOQ of 2.3 × 10^9^ particles mL^−1^, and a repeatability (_av_CV%) of 3.1%. A second calibration curve was measured using conventional magnetic beads (MBs, Figure S6, Supporting Information) to compare the performance of the two strategies. Already at these early stages of development, the coacervate‐based approach achieved comparable LOD of the state‐of‐the‐art beads (2.0 × 10^8^ particles mL^−1^ bead; LOD = 6.5 × 10^8^ particles mL^−1^ coacervate), indicating their applicability in urine samples, which contain on average 10^9^ particles mL^−1^.^[^ ^35^, ^36^, ^37^ ^]^ However, the bead‐based assay displayed a narrower dynamic range, as shown in Figure S6D, and lower repeatability (_av_CV% = 12%) compared to the coacervate‐based assay (_av_CV% = 3.1%), in addition to requiring a longer experimental time. Next, we evaluated the applicability of the coacervate‐based approach to real matrices by analyzing a pooled donor sample of human urine. Due to the unavailability of synthetic or recombinant analogs of urinary EVs, we evaluated a dilution response trend by performing serial dilutions of human urine (from 1:5 to 1:100) and profiling CD9, a classical urinary EV signature monitored in clinical practice.^[^ ^37^, ^38^ ^]^ We determined the noise signal by performing a control experiment with non‐functionalized coacervates at the highest urine dilution factor (1:5) (Figure S6F, Supporting Information). We obtained signals that were significantly different from the noise level (subtracted from the data set) up to a sample dilution limit of 1:150 (Figure 6B). In a clinical setting, the high dilution factor achievable with the coacervate strategy is particularly beneficial when dealing with limited volumes of urine samples or when samples are too turbid or contain a high density of interferences that typically require dilution to limit the matrix effect.

Furthermore, we observed a dilution response trend with excellent repeatability of _av_CV% = 3% (Figure 6B). As a control, we tested the polymer modified only with the hydrophobic linker (DBCO) used for the click reaction and observed only minimal interaction with the urine sample (equal to 15% of the signal obtained with the peptide‐functionalized coacervate, Figure S6F,G, Supporting Information).

Importantly, when the same samples were measured using the bead‐based assay, we observed high standard deviations that made it difficult to establish a consistent dilution response trend and an appropriate working dilution factor within the investigated range (Figure 6C). Moreover, the coacervate‐based assay significantly simplified the pre‐analytical workflow and, in our experiments, reduced the overall analysis time from 17 to 4.5 h compared to conventional beads (Figure 6D). Furthermore, when compared with a total urinary exosome isolation kit, the coacervate‐based strategy achieves comparable recovery using a significantly smaller starting volume of urine (in our case 100 µL vs 5 mL) (Figure S7, Supporting Information), making it efficient when sample availability is limited. Moreover, the absence of a concentration step (15 X) minimizes the potential risks of EV aggregation. These improvements have important implications for potential clinical applications of the coacervate‐based approach.

## 3 Conclusion

In this study, we have demonstrated a method for the selective isolation of EVs from complex biofluids using phase‐separated zwitterionic coacervates functionalized with a membrane‐sensing peptide. These coacervates form over a wide range of pH values and salt concentrations, making the method applicable to all biofluids. Moreover, the coacervates exhibit antifouling properties, thereby minimizing non‐specific binding. We have showcased the potential of this technology for two applications: as a preparative step for downstream analysis, and as a “one‐pot” assay for EV biomarker profiling.

We showed that the method can recruit and release intact EVs from urine samples with high yield. Importantly, we demonstrated the ability to isolate EVs from lipoproteins directly from a human urine sample, a task that is challenging with conventional separation methods, achieving a depletion of lipoproteins above 83%. The approach is scalable and concentrates EVs during isolation, allowing direct profiling of EV biomarkers within the coacervates by appropriate staining. The readout is performed as a one‐pot assay using conventional flow cytometry commonly available in research and clinical laboratories. Furthermore, we have shown that the one‐pot assay significantly streamlines the workflow and increases the signal‐to‐noise ratio by minimizing non‐specific binding. Overall, we have proven the potential of phase‐separated zwitterionic coacervates for affinity‐based isolation of EVs from complex mixtures, with implications for both large‐scale manufacturing and the analysis of biological fluids in the context of liquid biopsy.

## 4 Experimental Section

### Chemicals and Reagents

Methacrylamide (MAm, 98%), mono‐2‐(methacryloyloxy)ethyl succinate (HEMA‐Succ., ≥95%), 4,4′‐azobis(4‐cyanovaleric acid) (ACVA), 4‐cyano‐4‐(phenylcarbonothioylthio)pentanoic acid (CPA, ≥97%), di(ethylene glycol) methyl ether methacrylate (95%), ethylene glycol methyl ether methacrylate (95%,), N‐(3‐sulfopropyl)‐N‐methacroyloxyethyl‐N,N‐dimethylammonium betaine (SB), ethanol (≥99.8%), 1,3‐propanediol cyclic sulfate (TMS, 98%), sodium acetate (≥99%), acetic acid (≥99%), dimethyl sulfoxide (DMSO for molecular biology, ≥99.9%), chloroform (≥99%), polyoxyethylene sorbitan monooleate (Tween‐20), sodium chloride (NaCl, ≥99.5%), sodium hydroxide (NaOH, ≥97% pellets), hydrochloric acid (HCl, ≥37%), ammonium hydroxide solution (28% v/v), citric acid monohydrate (≥98%), N‐(3‐Dimethylaminopropyl)‐N′‐ethylcarbodiimide hydrochloride (EDC, ≥98%), dibenzocyclooctyne‐amine (DBCO‐NH_2_, ≥94.5%), Sepahrose CL‐4B, 5 cSt silicone oil, bovine serum albumin (BSA), Tween‐20 and Sigmatrix urine diluent were all purchased from Sigma‐Aldrich (Merck, Germany). Acetonitrile (ACN, 99.99%) and 2‐(N‐morpholino) ethanesulfonic acid (MES) anhydrous (≥99.5%), were obtained respectively from Fisher Chemicals (Fair Lawn, NJ, USA) and PanReac AppliChem (Germany). Di‐sodium hydrogen phosphate dihydrate (Na_2_HPO_4_·_2_H_2_O, ≥99.5%), sodium phosphate monobasic dihydrate (NaH_2_PO_4_·_2_H_2_O, ≥99.5%), potassium chloride (KCl, ≥99.5%), potassium phosphate monobasic (KH_2_PO_4_, ≥99.5%), and sodium carbonate (Na_2_CO_3_, ≥99.5%) were acquired from VWR Chemicals (Germany). 1,2‐dioleoyl‐sn‐glycero‐3‐phospho‐L‐serine (sodium salt) – 18:1 PS (DOPS, ∼95%) and 1,2‐dimyristoyl‐sn‐glycero‐3‐phosphoethanolamine‐N‐(lissamine rhodamine B sulfonyl) (ammonium salt) – 14:0 Liss Rhod PE (≥99%) were provided by Avanti Polar Lipids (Alabaster, AL). 3‐octadecyl‐2‐[3‐(3‐octadecyl‐2(3H)‐benzoxazolylidene)‐1‐propenyl]‐perchlorate (DiO), Gibco PBS pH 7.4, anti‐human CD81 monoclonal antibody APC (mAb, clone 1D‐6‐CD81), anti‐human CD63 mAb PE (clone H5C6), anti‐human CD9 mAb FITC (clone SN4C3‐3A2), Mouse IgG1 kappa isotype control APC (clone P3.6.2.8.1), Exosome‐Human CD81 flow detection reagent (from cell culture), Pierce Universal Nuclease, MagnaBind Carboxyl Derivatized Beads (Cat. No. 21 353) and CML Latex Beads, 4% w/v, 5 µm (Invitrogen. Cat. No. C37255) were acquired from Thermo Fisher Scientific (Waltham, MA, USA). The Bk‐MSP peptide ligand (sequence: RPPGFSPFR‐(O_2_Oc)‐RPPGFSPFR‐K‐G‐(O_2_Oc)‐N_3_, MW = 2688.41 g mol^−1^), was synthesized according to a previously published procedure.^[^ ^18^ ^]^ Human Lipoprotein A SimpleStep ELISA Kit was obtained from Abnova (Taipei, Taiwan). Human urine collected from pooled donors (≥ 3 donors per pool) was purchased from Lee Biosolutions (St. Louis, MO, USA). All chemicals used were of analytical grade purity and they were used without further treatment. All the buffer solutions were prepared with Milli‐Q water (18.2 MΩ cm^−1^ resistivity) and filtered with a microporous filter (0.22 µm Millipore filter). 96‐well tissue culture test plates and 384‐well plates (MatriPlate clear bottom, 0.17 mm glass) were respectively acquired from TPP (Greiner, Kremsmuenster, Austria) and from Brooks (Chelmsford, MA).

### Multiblock Zwitterionic (ZW) Polymer Synthesis

The copolymer was synthesized by two‐step reversible addition‐fragmentation chain‐transfer (RAFT) polymerization according to a previously established protocol.^[^ ^15^ ^]^ Briefly, sulfabetaine methacrylate (ZB) and N‐(3‐sulfopropyl)‐N‐methacroyloxyethyl‐N,N‐dimethylammonium betaine (SB) monomers were first copolymerized using the initiator (ACVA) and the RAFT agent (CPA) at a molar ratio of 3:1. The monomer‐to‐CPA molar ratio, which represents the degree of polymerization of an individual monomer i (DPi) was set equal to DP_ZB_ = 140 and DP_SB_ = 60. Specifically, 2.88 g SB, 7.10 g of ZB, 48 mg of CPA, and 16.1 mg ACVA were dissolved in 76.9 g of acetic acid buffer pH 4.5 and 15.1 g of ethanol and poured into a septum‐sealed flask. The synthesis and purification were performed as described previously.^[^ ^15^ ^]^ Subsequently, the polymeric scaffold was enriched with carboxylic groups by a sequential RAFT polymerization. To this aim, 198 mg of HEMA‐Succ was added to the initial reaction mixture, corresponding to DP_HEMA‐Succ_. = 5, and the same synthesis pathway was repeated. An aliquot of the reaction mixture was analyzed before and after the reaction completion using nuclear magnetic resonance (^1^H‐NMR). The samples were dried under nitrogen, dissolved in 3 M NaCl D_2_O, and analyzed on a 400 MHz NMR spectrometer (Bruker).

### Peptide‐Polymer Functionalization Procedure

10 mg of polymer (DP_ZB_ = 140 ZB; DP_SB_ = 60, HEMA‐Succ., MW = 58 000 g mol^−1^) was dissolved in 210 µL of MES buffer (composition: 100 mM MES, 1 M NaCl, pH 6) and stirred at room temperature (RT) until complete dissolution. The functionalization of the polymeric chains with an amphipathic peptide was achieved following a two‐step protocol. In the first step, DBCO‐NH_2_ (10 µL, 15 g L^−1^ diluted in DMSO) was covalently coupled to the polymer solution by activation of carboxylic groups with 5 mg of NHS and 3 mg of EDC. Then, the pH of this solution was raised to pH 7.4 using 1 m Na_2_CO_3_ (pH 11, 13.5 µL) before quickly adding DBCO‐NH_2_. The amine‐coupling reaction proceeded for 1 h at room temperature under stirring (600 rpm). Afterward, the modified polymer was washed with 15 mL of Milli‐Q water and centrifugated at 7000 rpm for 30 min. The recovered pellet was resuspended in 200 µL of 10 mM PBS pH 7.4 with 1.2 M NaCl (composition: 1.2 M NaCl, 28 mM NaH_2_PO_4_, 72 mM Na_2_HPO_4_) and 30 µL of peptide‐N_3_ (46.7 g L^−1^ in DMSO) was added. The second step involved the alkyne (DBCO)‐azide cycloaddition (copper‐free click chemistry reaction), which was left to react overnight at 25 ± 0.5 °C under orbital stirring (800 rpm). Unreacted components were separated from the polymeric scaffold by performing two washing steps with Milli‐Q water (1.2 mL), each followed by centrifugation (20 min at 11 000 rpm). Finally, the peptide‐conjugated polymer was resuspended in PBS pH 7.4 with 1.2 M NaCl to achieve a theoretical concentration of 40 g L^−1^ and the final batch was stored at 4 °C. The yield of the functionalization process was assessed using size‐exclusion chromatography (SEC) coupled with online fluorescence (1260 Infinity II) and UV–vis detection (Agilent 1100 multi‐wavelength diode‐array detector). Non‐functionalized polymer, functionalized polymer, and peptide‐N_3_ samples were injected (15 µL) in an Agilent HPLC system coupled with a Superdex 75 10/300L column (Cytivia, USA),

And eluted in 10 mM PBS at pH 7.4 and with 1.2 M NaCl at a constant flow rate of 0.8 mL min^−1^ over 40 min. The excitation (λ_Ex_) and emission (λ_Em_) wavelengths for L‐phenylalanine detection were set respectively at 260 ± 20 nm and 300 ± 70 nm (gain 14).

### Coacervate Fusion Events

The fusion events of the coacervates were monitored using a Nikon Ti2 Eclipse inverted microscope, equipped with a 60x/1.4 NA oil immersion objective (Nikon), a LED light source (Omicron, LEDhub), and a CCD camera (Andor, Zyla sCMOS 4.2P‐CL10). An aqueous solution of 2 g L^−1^ polymer at 100 mM of NaCl was incubated in a 384‐well plate, pre‐treated with 1% BSA aqueous solution to avoid droplet wetting. Images were acquired in brightfield and fluorescence every 30 milliseconds.

### Dependence of Phase Separation on Salt Concentration and pH

Functionalized polymer at a constant concentration of 0.8 g L^−1^ was mixed with increasing NaCl concentrations (10, 50, 100, 150, 200, and 300 mM). 40 µL of each solution was incubated at room temperature in a 384‐well plate, which was then sealed with 10 µL of silicon oil to prevent evaporation and covered with aluminum foil. The glass surface of the micro‐welled plate was pre‐treated by incubating 100 µL/well of 1% BSA aqueous solution for 2 h at room temperature, followed by a thorough rinse with Milli‐Q water. The same experiment was repeated to evaluate the influence of pH on phase separation, keeping a constant NaCl concentration of 100 mM while varying the pH from 3.5 to 8.5. Brightfield imaging was carried out on a Nikon Ti2 Eclipse inverted microscope, equipped with a 60x/1.4 NA oil immersion objective.

### Preparation and Characterization of Fluorescent Liposomes

Fluorescent liposomes were prepared using the thin‐film hydration method followed by extrusion according to a previously described procedure.^[^ ^17^ ^]^ Briefly, a lipid film composed of 18:1 PS (DOPS) and 14:0 Liss Rhod PE (200:1 molar ratio) was formed in a round‐bottom flask by removing chloroform first under a nitrogen stream for 2 h and then under vacuum overnight. The dry lipid film was hydrated with 1 mL of 10 mM PBS pH 7.4 and gently stirred to obtain heterogeneous liposomes. This lipid suspension was then extruded 20 times through a Whatman polycarbonate hydrophilic membrane with a 0.1 µm pore size (Nucleopore, Cytivia). DiO‐labeled liposomes were prepared in a similar manner, substituting 14:0 Liss Rhod PE with DiO lipophilic tracer. Potential free dye was removed by size‐exclusion chromatography (SEC) using a 10 cm gravity column (Econo‐Pac, Biorad, USA) packed with Sepharose CL‐4B (a cross‐linked 4% agarose matrix with an average pore size of 100 µm). Fractions were eluted with 1 mL of 10 mM PBS pH 7.4 (Gibco). Liposome‐enriched fractions (500 µL) were collected and analyzed using dynamic light scattering (DLS) with a Zetasizer Nano‐ ZS (Malvern Instruments, UK), and nanoparticle tracking analysis (NTA) with a ZetaView NTA instrument (Particle Metrix, Germany) equipped with a CMOS camera and a 405 nm laser beam. *NTA* measurements: The NTA instrument was calibrated according to the supplier's instructions using a 1:2500 dilution of polystyrene beads in PBS pH 7.4 (Gibco). Liposomes were then injected at 1:1000 dilution in PBS to achieve a concentration spanning from 10^6^ to 10^8^ particle mL^−1^. The Brownian movement of each particle was tracked in a frame‐by‐frame manner at 11 positions through the sample cell, with data acquisition performed at 30 frames per second for 60 s. Scattered signals were measured at a sensitivity of 89 and a shutter of 100. Data were analyzed using the ZetaView analysis software (ZetaView 8.04.02 SP2 – Particle Metrix).

### Production and Fluorescent Labeling of EVs Serived from HEK 293‐F Cells

HEK 293‐F cells were cultured following the procedure described by Paganini et al.,^[^ ^39^ ^]^ using a batch‐refeed system where cell bleeding and medium exchange were performed at regular intervals. The harvested medium (50 mL) was clarified by centrifugation at 3000 rcf for 15 min and by filtration through a 0.22 µm PES membrane (Greiner, Kremsmuenster, Austria). Subsequently, 25 U of Pierce Universal Nuclease was added to the medium and incubated for 2 h at room temperature. The medium was then concentrated to ≈500 µL using Amicon filters with a MWCO of 50 kDa (Millipore Corp.) before collecting the EV‐enriched fractions via SEC. EVs were characterized in terms of particles mL^−1^ using NTA and in terms of expression of biomarkers (tetraspanins) on the surface using flow cytometry. Fluorescent EV labeling was carried out largely following the procedure reported by Chao et al.^[^ ^40^ ^]^ Specifically, EVs were diluted to 7.40 × 10^9^ particles mL^−1^ in an aqueous solution containing 15 mM NaCl, 0.004% Tween‐20, 2 µM of DiO, and incubated for 30 min at 37 °C under stirring at 300 rpm, followed by centrifugation at 5000 rcf for 20 min at 4 °C to remove cell debris and/or aggregates.

### Confocal Microscopy Analysis

Confocal imaging was performed on a Leica SP8‐AOBS‐CARS laser confocal microscope equipped with a 63x/1.40 oil HC PL APO CS2 objective, an Orca‐Flash4.0 sCMOs camera (Hamamatsu, USA), and AOBS laser system (HyD detector).

### Evaluation of Vesicle Uptake Via Size‐Exclusion Chromatography (SEC)

The recruitment of DiO‐labeled liposomes was performed by incubating 2.5 g L^−1^ of functionalized polymer with a fixed amount of DiO‐liposomes (5.5 × 10^9^ particles mL^−1^) in PBS at pH 7.4 and 100 mM NaCl for 20 min at 10 °C under a constant shaking at 300 rpm. 40 µL of the mixture was deposited into four different wells of a 384‐well plate, which were then covered with aluminum foil. After 1 h, the samples were centrifuged for 15 min at 4000 rpm and T = 4 °C, and 20 µL of supernatant was collected from each well, pooled, and analyzed via SEC. All measurements were conducted by injecting 15 µL of sample into an Agilent HPLC system combined with a Tricorn‐5/100 column (Cytivia, USA) packed with a Sepharose‐CL4B resin. The samples were isocratically eluted with PBS at pH 7.4 (140 mM NaCl, 2.68 mM KCl, 3.56 mM NaH_2_PO_4_, 6.44 mM Na_2_HPO_4_) and at a constant flow rate of 0.1 mL min^−1^ over 60 min. The λ_Ex_ and λ_Em_ wavelengths were set at 480 ± 40 nm and 520 ± 40 nm, respectively. EVs derived from HEK 293‐F were spiked at 1.2 × 10^10^ particles mL^−1^ in artificial urine, and incubated with 2.7 g L^−1^ functionalized polymer in a phosphate buffer solution containing 80 mM NaCl. The pH of the solution was adjusted to obtain an alkaline environment using sodium hydroxide or ammonium hydroxide solution (28% v/v). After 2 h of incubation, the intrinsic fluorescence signal of the total protein content was measured at the wavelengths, λ_Ex_ = 280 ± 20 nm and λ_Em_ = 350 ± 20 nm. The same protocol used for EVs from HEK 293‐F was applied to explore the recruitment of EVs directly from a human urine sample. To this aim, human urine diluted 1:5 was incubated with the functionalized polymer for at least 3 h at 10 °C and 800 rpm stirring speed, followed by centrifugation to collect the supernatant, which was then stained with 0.6 nM anti‐CD9‐FITC. The staining of the specific biomarker was performed overnight at 4 °C under orbital stirring at 800 rpm. All the stained samples, together with a control of 0.6 nM anti‐CD9 Ab in PBS, were analyzed at the wavelengths λ_Ex_ = 480 ± 30 nm and λ_Em_ = 520 ± 30 nm (gain 18).

### TEM Analysis

EVs were released by dissolving the dense coacervate phase in a high‐salt solution (10 mM PBS pH 7.4 with 600 mM NaCl and 500 mM MgCl_2_) before the analysis. 3.5 µL of sample was deposited onto a freshly glow‐discharged (25 mA; 30 s; PELCO EasiGlow) Cu 400 mesh carbon‐coated grid (Quantifoil) and incubated at room temperature for 30 s. Excess liquid was removed using filter paper, and the grids were briefly (ca. 5 s) washed with water and blotted. Subsequently, the grids were negatively stained twice for 30 s each using a 2% (w/v) aqueous uranyl acetate solution. Excess stain was removed by blotting with filter paper after each staining step. Finally, the grids were air‐dried, and TEM images were acquired using a Morgagni 268 electron microscope in bright field mode, operated at 100 kV, which is equipped with a CCD camera (1376 × 1032 pixels).

### Flow Cytometry Analysis of Liposomes

RhB‐labeled liposomes in buffer were calibrated using 0.8 g L^−1^ polymeric coacervate over a concentration range spanning from 3.9 × 10^7^ to 1.0 × 10^10^ particles mL^−1^, following the uptake procedure described for the SEC analysis. All the samples were deposited in a TPP 96‐well tissue culture plate and analyzed using the FACSymphony A5 flow cytometer (BD Biosciences) with BD FACSDiva software. The quantification of RhB‐labeled liposomes was performed on a FACSymphony A5, equipped with a yellow‐green laser (561 nm) and a PE filter (bandpass: 586/15 nm, gain = 1000). For all the measurements, the following setting was used: forward scatter (FSC) voltage = 400, side scatter (SSC) voltage = 320, sample flow rate = 2 µL s^−1^, sample volume = 10 µL, mixing volume = 100 µL, mixing speed = 180 µL s^−1^, number of mixes = 2, wash volume = 400 µL. After each sample injection, the system was washed with a high salt solution (1.2 M NaCl) to prevent carry‐over effects. The quantification of RhB‐Lip was performed by applying the gating strategy shown in Figure S8, Supporting Information. Specifically, a threshold of fluorescence was set to remove the presence of distinct fluorescent populations, potentially related to aggregate formation, which occurred at a very high RhB‐Lip. concentration (1.0 × 10^10^ particles mL^−1^). Data analysis was conducted using OriginLab 2022b and FlowJo Vx 10.0.8. The analytical figures of merit, LOD, and LOQ, were calculated with the following equations: LOD = 3σ × EC_50_/FL_max_ and LOQ = 10σ × EC_50_/FL_max_, where σ is the standard deviation of the mean signal of the blank solution. Control measurements were performed under the same conditions. Liposomes were also imaged using Amnis ImageStream X MkII, a flow cytometer that allows for the visualization of flowing objects through both brightfield and fluorescence imaging. Samples were analyzed directly from a 1.5 mL tube using a 60X magnification (providing a pixel size of 0.3 um^2^), a low flow rate/high sensitivity, and a manual stream (focus/centering) adjustment where necessary. Fluorescence and SSC were activated with lasers 488 nm and 785 nm, respectively. The ISX software on the instrument was configured as follows: brightfield (channels 1 and 9), PE channel 3 (bandpass filter: 577/35 nm), and scattering (channels 6 and 9). Each cell on ImageStream was simultaneously imaged in brightfield and fluorescence mode. Data processing was carried out using IDEAS software 6.3 (Amnis Corp.).

### Western‐Blot Analysis

5X Laemmli buffer was added to the HEK 293‐F cell samples (described in Figure 3), and they were heated at 95 °C for 5 min. Soluble proteins were first separated by SDS‐PAGE (4%–20%, Mini‐Protean TGX Precast protein gel, Bio‐Rad) and then transferred onto a PVDF membrane. The membrane was blocked to saturate nonspecific binding with 1% BSA in PBS‐0.05% Tween‐20 (PBS‐T) for 1 h at 37 °C. Membranes were then incubated overnight at 4 °C with anti‐ALIX (1:1000, Santa Cruz, CA, USA) and anti‐TSG101 (1:1000, Novus Bio, Centennial, CO, USA), diluted in 1% BSA PBS‐T. Afterward, the membranes were washed three times with PBS‐T and incubated with a rabbit anti‐mouse HRP‐conjugated secondary antibody (Jackson ImmunoResearch, Tucker, GA, USA) diluted 1:3000 in TBS‐T with 1% BSA for 1 h. After the washing steps, the signal was detected using Bio‐Rad Clarity Western ECL Substrate (Bio‐Rad) and imaged using a Chemidoc XRS+ (BioRad).

### Tetraspanin Profiling of EVs via Bead‐Based Assay

EVs derived from HEK 293‐F cells (2.60 × 10^8^ particles mL^−1^) and human urine were initially characterized to obtain an overview of the tetraspanin profile using a commercial bead‐based assay. The assay was performed following the manufacturer's instructions and a previously published protocol.^[^ ^22^, ^39^ ^]^ The final bead suspensions were resuspended in 1.5 mL (EVs HEK 293‐F) and 0.5 mL (human urine) of 10 mM PBS pH 7.4 with 0.1% BSA and then analyzed using Cytoflex S flow‐cytometer (Beckman Coulter, USA). The gains of the forward scattering (FSC), side scattering (SSC, bandpass filter: 488/8 nm), and violet SSC (bandpass filter: 405/10 nm) were set at 199, 48, and 50, respectively. In this case, three different fluorescent monoclonal antibodies (mAbs) were used: anti‐CD63 PE (bandpass filter: 585/42 nm, gain = 3000), anti‐CD81 APC (bandpass filter: 660/10 nm, gain = 3000), IgG1 isotype control APC, and anti‐CD9 FITC (bandpass filter: 525/40 nm, gain = 3000). All the samples were analyzed at a medium flow rate of 30 µL min^−1^ for 60 s after mixing for 3 s (backflush 3 s).

### Flow Cytometry Analysis of EVs

2 g L^−1^ of functionalized polymer in 10 mM phosphate solution at pH 10 (with 100 mM NaCl and 0.1% BSA) was incubated with different concentrations of EVs from HEK 293‐F cells (range: 8.1 × 10^7^ to 5.2 × 10^10^ particles mL^−1^) spiked in artificial urine for 3 h at 10 °C under constant stirring at 800 rpm. Then, 3 nM anti‐CD81‐APC Ab was added to each sample. The solutions were further incubated for 1.5 h at 25 °C (800 rpm) and measured using a FACSymphony A5 flow cytometer with the same settings as liposomes.

### Comparison Between Bead‐ and Coacervate‐Based Strategy for EV Isolation as Preparative Step

MagnaBind Carboxyl Derivatized Beads (Cat. No. 21 353), CML Latex Beads, 4% w/v, 5 µm (Invitrogen. Cat. No. C37255), and the polymer were functionalized with Bk‐MSP largely following the procedure described above. DBCO‐NH_2_ was covalently coupled to the beads/polymer by activation of carboxylic groups with 50 µL of 18 mM of EDC. Then, beads and coacervates (V = 400 µL) were incubated with 1.2 × 10^10^ particles mL^−1^ EVs from HEK‐293F spiked in artificial urine, before applying the same procedure described in the previous paragraphs. The beads, after the incubation step, were washed three times with PBS pH 7.4 following the manufacturer's instructions. All the pellet fractions were resuspended in 600 mM NaCl and 500 mM MgCl_2_ and incubated for 1 h at 25 °C and 800 rpm to release EVs from the peptide ligand (Bk‐MSP). The dissolved coacervates and the supernatants collected from the beads were analyzed with a flow cytometer (Cytoflex S) to evaluate the presence of EV‐specific biomarkers (CD63 and CD81) using an anti‐CD81‐bead‐based immunoaffinity assay (as described above). The same protocol was used for the direct analysis of human urine samples, where the expression of CD9 and CD63 was evaluated using a flow cytometer to evaluate.

### Comparison Between Bead‐ and Coacervate‐Based Strategy for Direct Flow‐Cytometry Analysis

Peptide‐functionalized magnetic beads and functionalized coacervates were incubated with serial concentrations of EV HEK‐293F spiked in artificial urine ranging from (8.1 × 10^7^ to 5.2 × 10^10^ particles mL^−1^). The incubation step was carried out at 10 °C and 800 rpm stirring speed, for 3 h with coacervates and overnight with beads, and then EVs were stained with 3 nM anti‐CD81 Ab (3 nM). After each step, the bead‐based strategy required intensive washing steps, which were not necessary for the coacervate‐based assay (as illustrated in Figure 6D). For the analysis of the human urine sample with both strategies, serial dilutions of the sample were performed in buffer (from 1:5 to 1:100) before staining with 0.3 nM anti‐CD9 Ab. The measurements were conducted on a FACSymphony A5 flow cytometry (blue laser 488 nm, FITC filter bandpass: 530/30, gain = 1000) using the same setting previously described.

### Statistical Analysis

All quantitative data are presented as mean ± SD from three replicates and analyzed using OriginLab 2022b. The data were normalized by dividing each value by the maximum value in the dataset. The significance of the results when comparing three different approaches was evaluated using a paired‐sample *t*‐test (effect size: Cohen's d). The results revealed a significantly large difference between the three approaches (ZW coacervates, MBs, LBs). The data were analyzed as two groups (ZW coacervates vs MBs, ZW coacervates vs LBs) for both biomarkers investigated (CD63/CD81 and CD9/CD63), with *p *< 0.01. The normality of the data was assessed using the Shapiro‐Wilk test before applying the *t*‐test.

Flow cytometer data were analyzed using FlowJo Vx 10.0.8.

## Conflict of Interest

P.A. and C.P. has filed PCT/EP2021/08 4452 patent application titled “Programmable polymer droplets and associated uses”. A.G. and M.C. have filed PCT/IB2020/05 8284 patent application titled “Conjugates composed of membrane‐targeting peptides for extracellular vesicle isolation, analysis and their integration thereof”.

## Supporting information



Supporting Information

## Data Availability

The data that support the findings of this study are available from the corresponding author upon reasonable request.
