# Supplementary material for: Affinity‐Based Isolation and One‐Pot Analysis of Extracellular Vesicles from Biofluids Using Phase Separated Zwitterionic Coacervates
Source: Adv Sci (Weinh). 2025 Apr 15;12(20):2411653. doi: 10.1002/advs.202411653 (PMC12120729; doi:10.1002/advs.202411653)
Supplement: Supplementary file 1 — Supporting Information [file ADVS-12-2411653-s001.docx]

Supporting Information

**Affinity-based isolation and one-pot analysis of extracellular vesicles from biofluids using phase separated zwitterionic coacervates**

*Francesca Torrini^1^, Roberto Frigerio^1,2^, Jonathan Garlipp^1^, Philippe Lenzen^1^, Karl Normak^1^, Carolina Paganini^1^, Marina Cretich^2^, Alessandro Gori^2^, Paolo Arosio^1,*^*

^1^Department of Chemistry and Applied Biosciences, Institute for Chemical and Bioengineering, ETH Zurich, Zurich, 8093, Switzerland

^2^Consiglio Nazionale delle Ricerche, Istituto di Scienze e Tecnologie Chimiche “Giulio Natta” (SCITEC), 20131 Milan, Italy

*E-mail: [paolo.arosio@chem.ethz.ch](mailto:paolo.arosio@chem.ethz.ch)


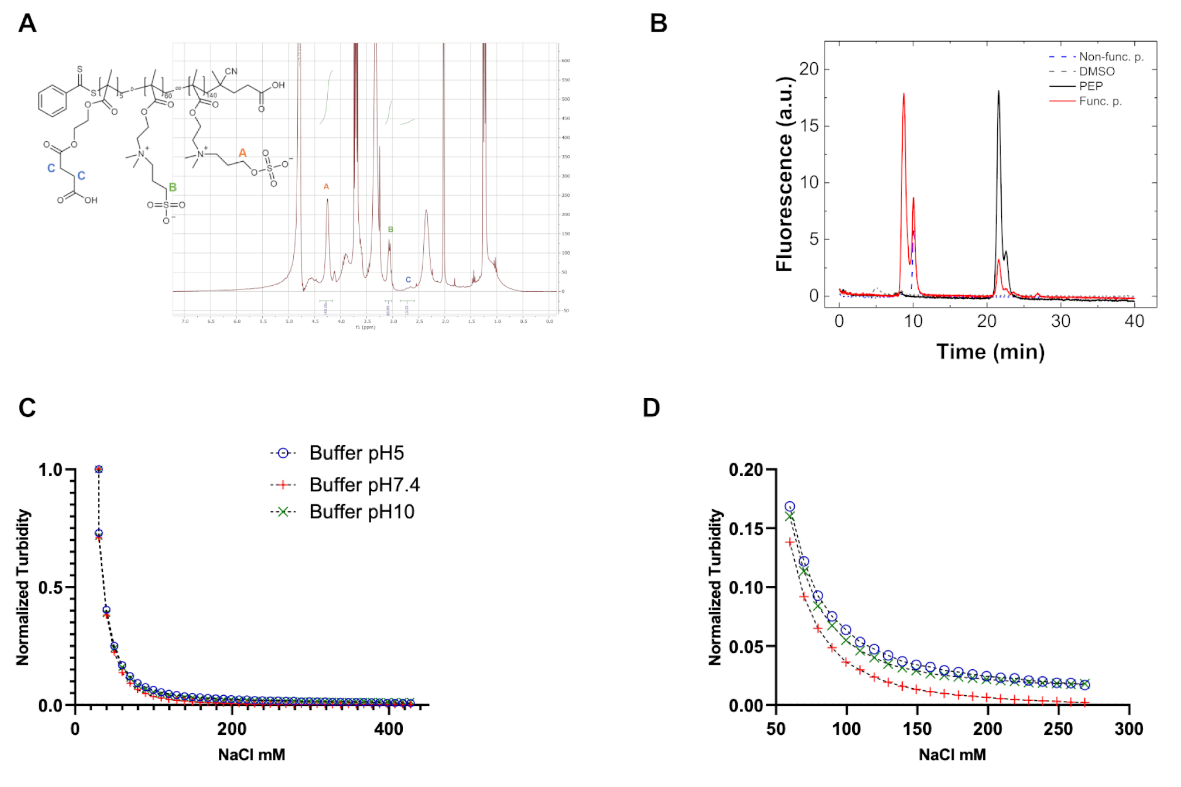


**Figure S1.** A) Representative ^1^H-NMR spectrum of the zwitterionic polymer (DP_ZB_ = 140 and DP_SB_ = 60) enriched with carboxyl groups in the backbone by performing a sequential RAFT polymerization using mono-2-(methacryloyloxy)ethyl succinate (HEMA-Succ., ≥95%). The spectrum was recorded after 24 hours on a Bruker 400 MHz spectrometer using 1M NaCl deuterium oxide as solvent. B) Representative SEC elution profiles of functionalized polymer (red line), non-functionalized polymer (blue dashed line), peptide-N_3_ (black line), and DMSO (10 % v/v) (gray dashed line). The chromatogram of the free peptide is characterized by a single fluorescent peak with a retention time of 21.6 minutes associated with phenylalanine residues (black line). A significant decrease of this peak was observed for the sample corresponding to the peptide linked to the polymeric scaffold (red line), indicating a substantial consumption of the peptide during the reaction. Correspondingly, a second fluorescent peak appears in the chromatogram of the functionalized polymer with a retention time of about 10 minutes, due to the presence of phenylalanine residues in the polymeric backbone after functionalization. The concentration of the free peptide after functionalization was calculated using the following formula: [peptide-N_3_]_unknown_= (peak_area_unknown_/response factor_peptide-N3_). The response factor_peptide-N3_ (RF) is defined as the ratio of the obtained response to the known concentration of peptide-N3, calculated with the formula RF = Peak_Area _peptide-N3_/[peptide-N_3_] mmol L^-1^. Functionalization efficiency (%) was calculated as follows: (C_functionalized_ [mmol L^-1^]/C_initial_ [mmol L^-1^]) × 100, where C_initial_ is the initial concentration of the peptide added to the polymer, C_functionalized_ = C_initial_ - C_free_, and C_free_ is the remaining concentration of free peptide after the functionalization process. We obtained a functionalization efficiency of 80%. C-D) Determination of the pH dependence of phase separation by evaluating the critical salt concentration (CSC) required to suppress coacervation. 1 g L^-1^ functionalized polymer was dissolved in three different 10 mM phosphate solutions at pH 5, 7.5, and 10. Light scattering measurements were performed on a Labbot instrument (Probation Labs Sweden AB, Sweden) by varying NaCl concentrations (40-400 mM) through a sequential and automatic titration with a 5 M NaCl solution. The phase separation behavior was consistent across all pH conditions tested within 40-200 mM NaCl (zoomed range in the D panel).


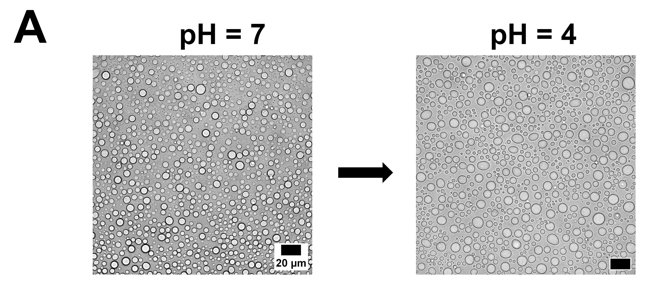


**Figure S2.** Representative brightfield microscopy images of 1 g L^-1^ polymer solution with 100 mM NaCl subjected to a sequential pH variation from pH = 7 to pH = 4.

**
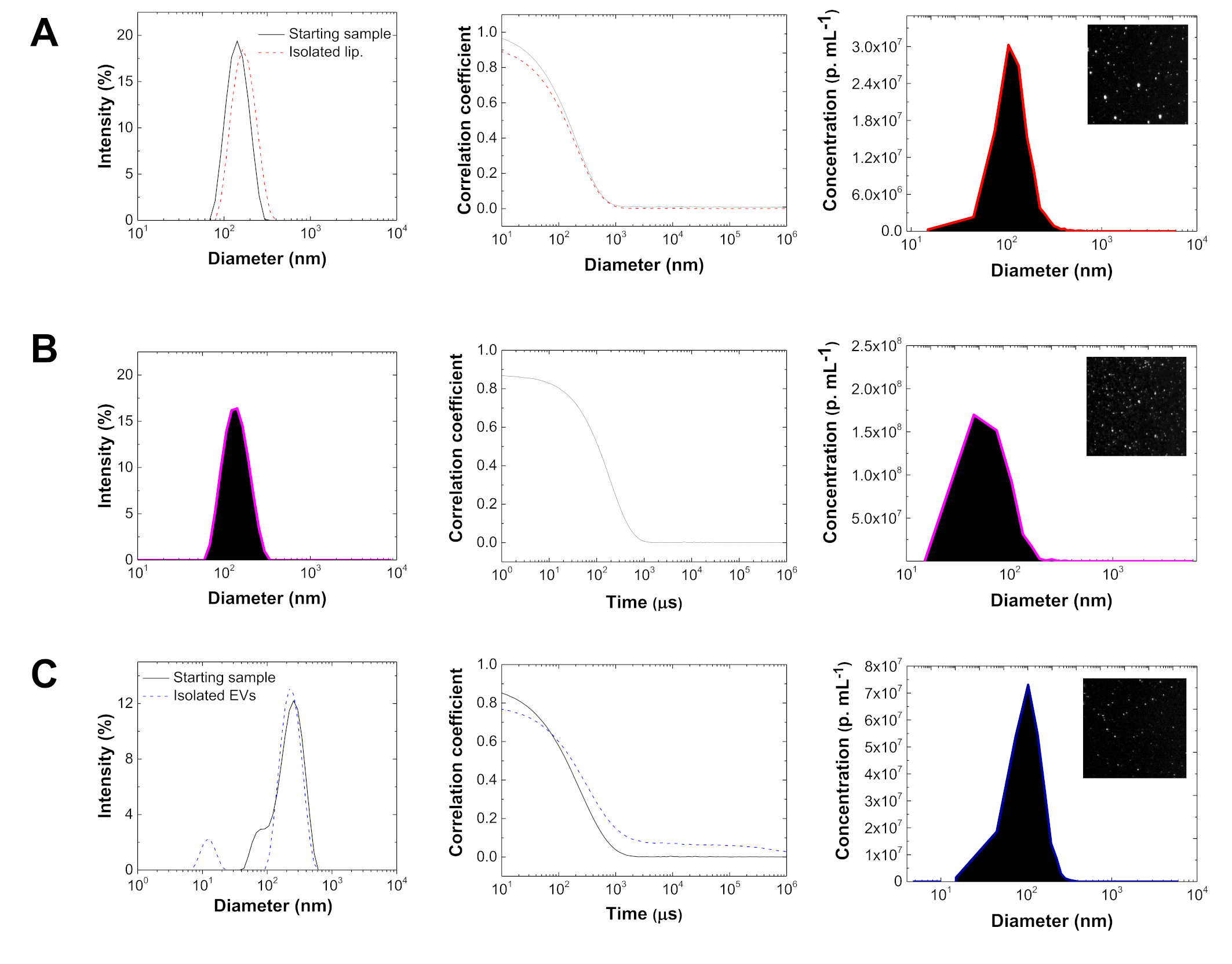
**

**Figure S3.** From left to right: DLS size distribution and autocorrelation curves obtained on samples before and after the isolation process. Nanoparticle tracking analysis (NTA) was performed on all the samples diluted 1:1000 in PBS pH = 7.4. A) DiO-labeled liposomes (diameter = 109 ± 4 nm); B) RhB-labeled liposomes (diameter = 100 ± 3 nm); C) EVs derived from HEK 293-F (diameter = 188 ± 9 nm).


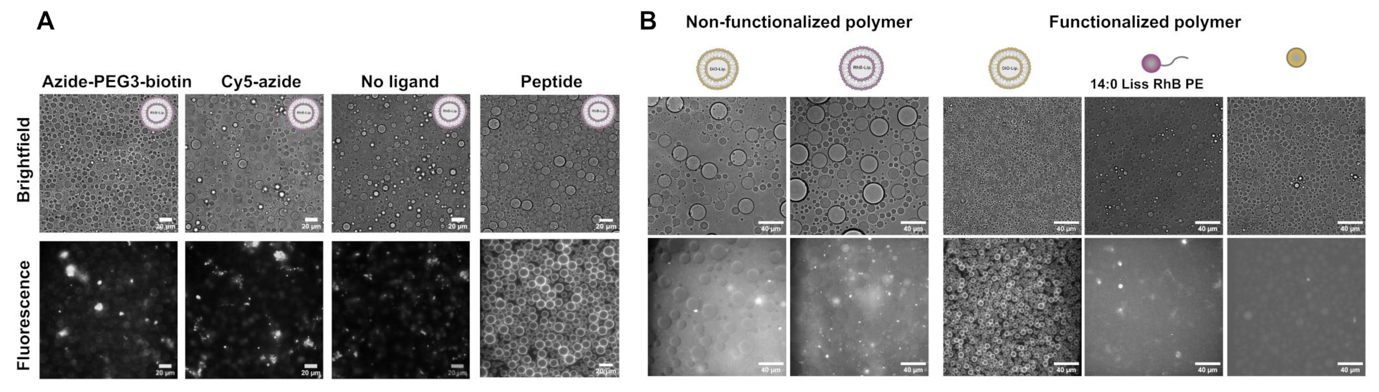


**Figure S4.** A) Brightfield and fluorescence images of coacervates formed by 0.8 g L^-1^ of functionalized polymer with different ligands, from left to right: Azide-PEG3-biotin, Cy5-azide, free alkyne without azide ligand, and the peptide-N_3_ (all these ligands were added during the functionalization procedure at a fixed concentration of 19.4 mmol L^-1^, see experimental section); in the presence of 1.3 × 10^10^ particles mL^-1^ RhB-liposomes in PBS pH 7.4 with 100 mM NaCl (scale bar: 20 µm). B) Right: brightfield and fluorescence images (scale bar: 40 µm) of coacervates formed by 0.8 g L^-1^ non-functionalized polymer in the presence of DiO-liposomes (3.3 ×10^10^ particles mL^-1^) and RhB Liposomes (1.3 × 10^10^ particles mL^-1^). Left: brightfield and fluorescence images of coacervates assembled by functionalized polymer in the presence of DiO-liposomes, 14:0 Liss RhB PE, and DiO, respectively.


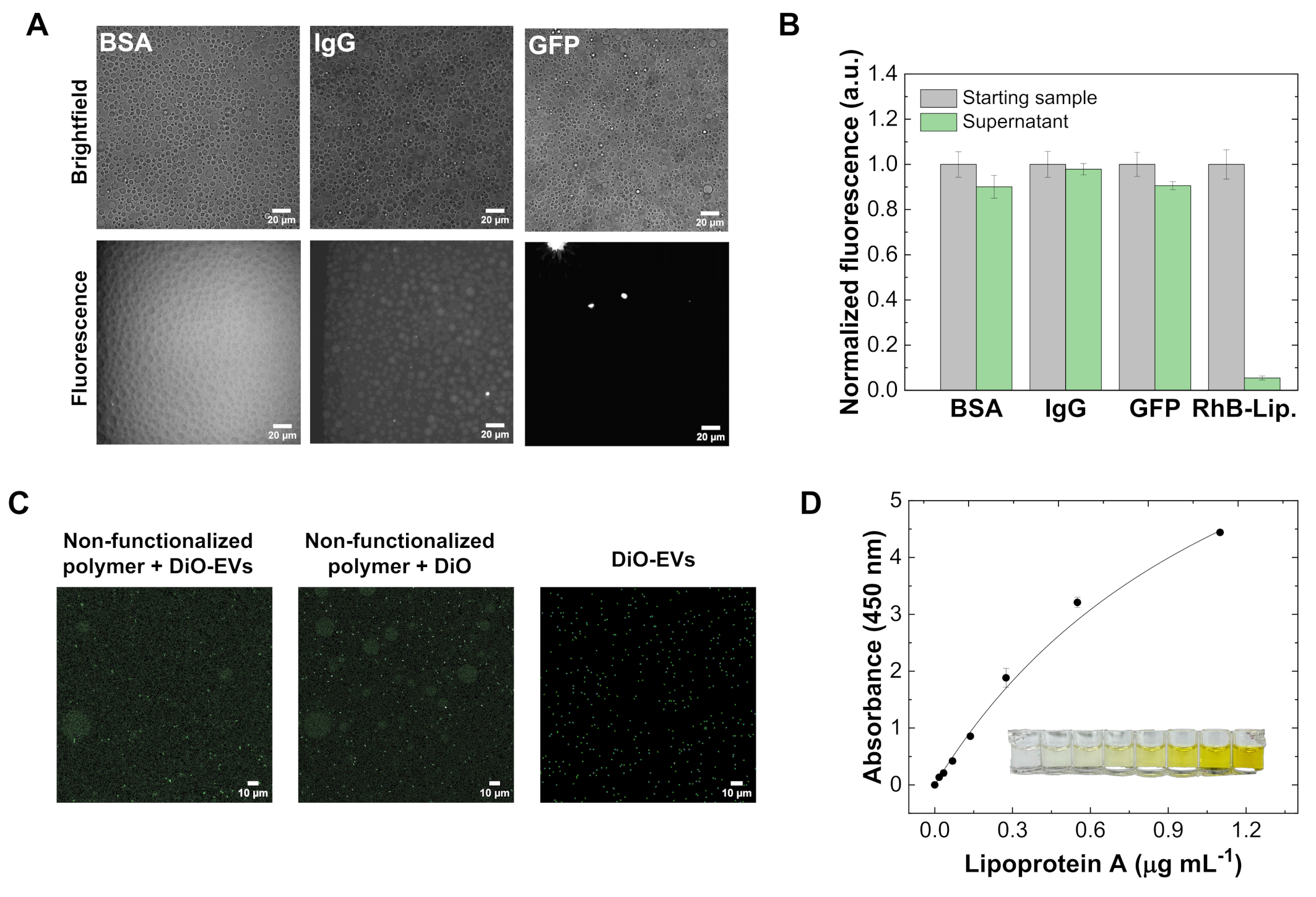


**Figure S5.** A) Comparison of fluorescence and brightfield images of coacervates formed by 0.8 g L^-1^ of functionalized polymer in the presence of 500 nM of BSA, IgG, or GFP. B) Assessment of binding by comparing the normalized fluorescence signals of BSA, IgG, GFP, and RhB-liposomes samples in the original samples (grey bars) and in the supernatants collected post uptake (green bar). As expected, we observed significant recruitment of liposomes and negligible uptake of the other biomolecules. Each bar indicates the normalized mean fluorescence (FL_mean_) ± standard deviation (SD) recorded in triplicates. C) Confocal microscopy images of non-functionalized polymer (3.3 g L^-1^) with DiO-EVs (9 × 10^8^ particles mL^-1^), DiO (2 µmol L^-1^), and DiO-EVs spiked in artificial urine. D) Calibration curve of the commercial SimpleStep ELISA kit for the detection of human lipoprotein A, where each point is representative of three measurements (Abs 450nm_mean_ ± SD).


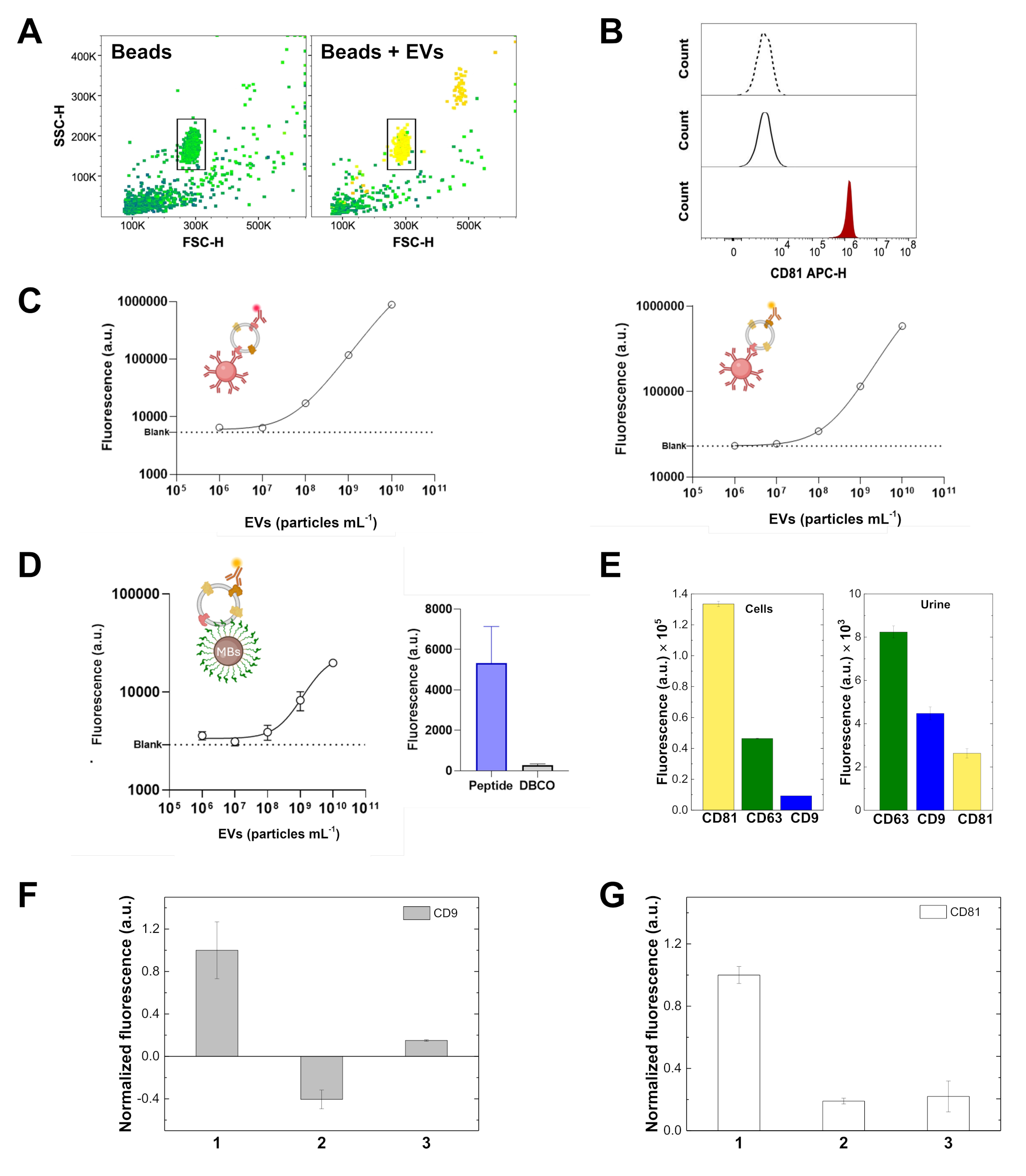


**Figure S6.** Bead-based flow cytometry assay for EV analysis. A) Representative 2D dot plots of forward scattering signal (FSC) vs. side scattering signal (SSC) of immunoaffinity based Dynabeads^TM^ (Exosome-Human CD81 flow detection reagent, see experimental section) in the absence and presence of EVs derived from HEK 293-F labeled with anti-CD81 antibody-APC. B) Stacked median fluorescence intensity (MFI) histograms of Dynabeads^TM^ in the absence (dashed line) and presence of EVs (HEK 293-F) labeled with an IgG1 isotype control-APC (black line) and anti-CD81 Ab-APC (red bell curve). C) Calibration curves of EVs in the range spanning from 1.2 × 10^6^ to 1.2 × 10^10^ particles mL^-1^. EVs were captured on Dynabeads^TM^ functionalized with anti-CD81 Ab, while anti-CD81 Ab-APC (left) and anti-CD63 Ab-PE (right) were used as signal reporters. Each point represents MFI_mean_ ± SD from triplicates. D) (left) EV calibration curve in the range of 1.2 × 10^6^ to 1.2 × 10^10^ particles mL^-1^ obtained using commercial carboxylate beads (MagnaBind^TM^) functionalized with the amphipathic peptide as the primary receptor and anti-CD81 Ab as the secondary receptor. Right: bar chart comparing the signals acquired in the presence of the coacervates functionalized with either the peptide or DBCO, a moderately hydrophobic molecule used as a linker in the click-chemistry reaction. Bars report FL_mean_ ± SD from triplicates. E) Histograms reporting the signal (MFI_mean_ ± SD) obtained with a standard bead-based assay for flow cytometry on 1.2 × 10^10^ particles mL^-1^ EVs HEK 293-F and human urine. This analysis provides information on the expression level of specific biomarkers (CD81, CD63, and CD9). Data were subtracted from the blank sample lacking EVs. F-G) Bar charts comparing the signals (MFI_mean_ ± SD) acquired for human urine (diluted 1:10) and artificial urine spiked with EVs HEK 293-F (5.2 × 10^10^ particles mL^-1^) supplemented with coacervates functionalized with the amphipathic peptide (1), not-functionalized coacervates (2), and functionalized coacervates with DBCO (3) using as signal reporters either Anti-CD9 Ab (F) or Anti-CD81 (G).





**Figure S7.** Comparison between total exosome isolation reagent and coacervate-based strategy for EV isolation. EVs from human urine sample were isolated using a precipitation-based total exosomes kit (Invitrogen, catalog number: 4484452) and the coacervate-based strategy developed in this study. Following the manufacture’s protocol for the kit, EVs were isolated from a starting volume of 5 mL clarified human urine, yielding a recovery volume of 0.3 mL in 10 mM PBS pH 7.4, corresponding to a concentration factor of 15X. The coacervate-based strategy was performed using 100 µL of human urine, with the final pellet resuspended in 150 µL of buffer (600 mM NaCl and 500 mM MgCl_2_). In both cases, the recovered pellets were dissolved in the appropriate buffer and analyzed with the flow cytometer (Cytoflex S) to characterize the specific biomarkers CD9 and CD63. The bar graph indicates the MFI_mean_ associated with the tetraspanins (CD63 and CD9) present on urinary EVs after being isolated with the commercial kit and the coacervate-based strategy, compared to the EV amount in the initial sample. All data were processed for their respective concentration factors.


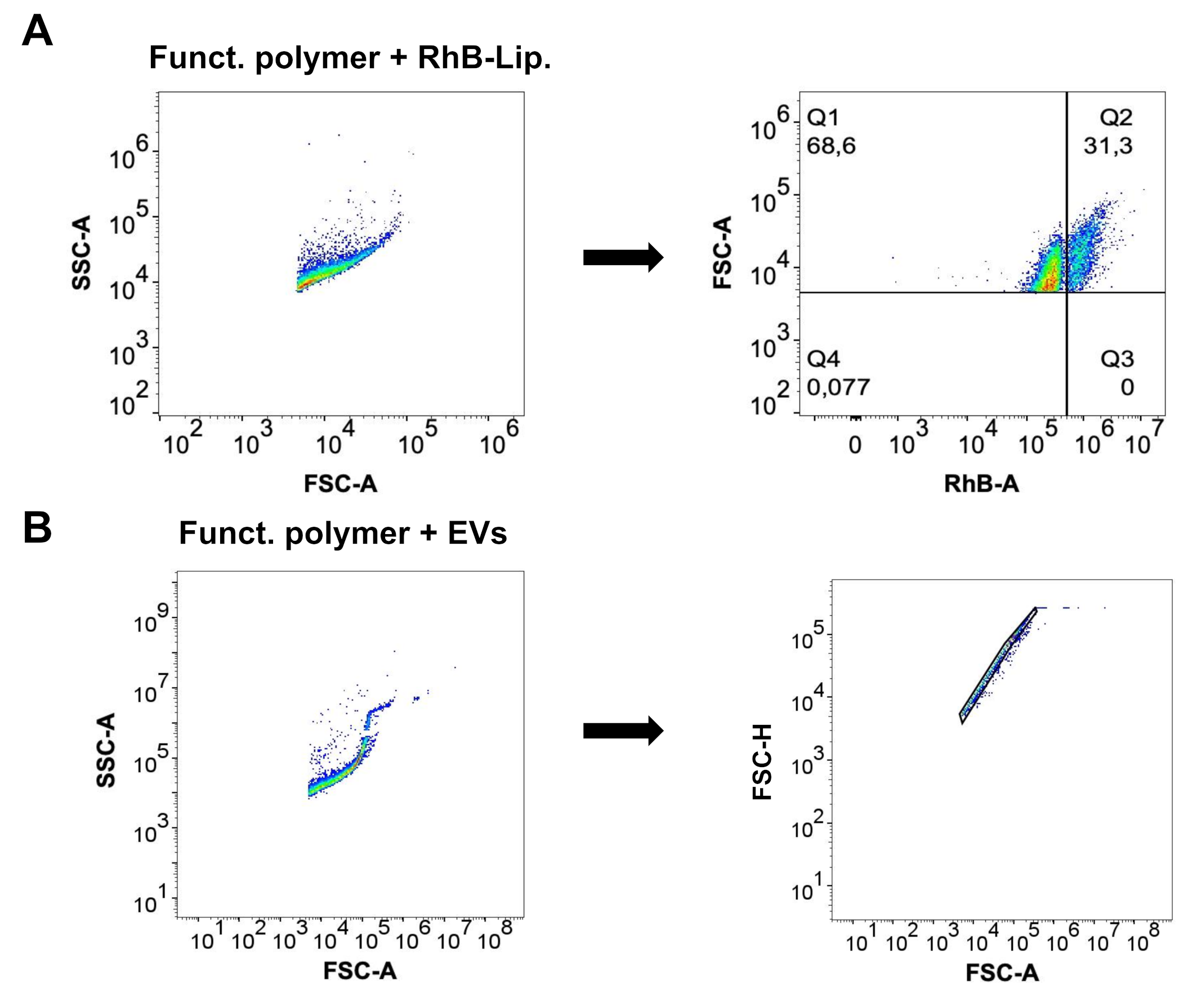


**Figure S8.** Representative 2D dot plots of forward light scattering (FSC-Area) vs. side light scattering (SSC-Area) of coacervates in the presence of RhB-Lip. and EVs from HEK 293-F (panels on the left). Panels on the right show the gating strategy (see experimental section) used for the flow cytometry detection of RhB-Lip (A panel illustrate FSC-A vs. fluorescence intensity) and EVs from HEK 293-F (B panel illustrate FSC-A vs. FSC-H), respectively.
